# Supplementary material for: Selection and hybridization shaped the rapid spread of African honey bee ancestry in the Americas
Source: PLoS Genet. 2020 Oct 19;16(10):e1009038. doi: 10.1371/journal.pgen.1009038 (PMC7595643; doi:10.1371/journal.pgen.1009038)
Supplement: S2 Table — Model rankings between logistic cline fits for genome-wide scutellata (A) ancestry predicted by climate and distance variables. (PDF) [file pgen.1009038.s002.pdf]

S2 Table. Cline model comparison. Model rankings between logistic cline fits for genome-wide *scutellata* (A) ancestry predicted by climate and distance variables.

	predictor	df.residual	deviance	dAIC	weight
1	Latitude	311	2.69	0.00	1.00
2	Mean temperature	311	3.63	93.60	0.00
3	Mean temperature of coldest quarter	311	5.25	208.60	0.00
4	Minimum temperature of coldest month	311	7.04	300.70	0.00
5	Distance to Sao Paulo	311	9.55	396.10	0.00
6	Annual precipitation	311	15.21	541.80	0.00
