# Supplementary figures and images for: Selection and hybridization shaped the rapid spread of African honey bee ancestry in the Americas
Source: PLoS Genet. 2020 Oct 19;16(10):e1009038. doi: 10.1371/journal.pgen.1009038 (PMC7595643; doi:10.1371/journal.pgen.1009038)

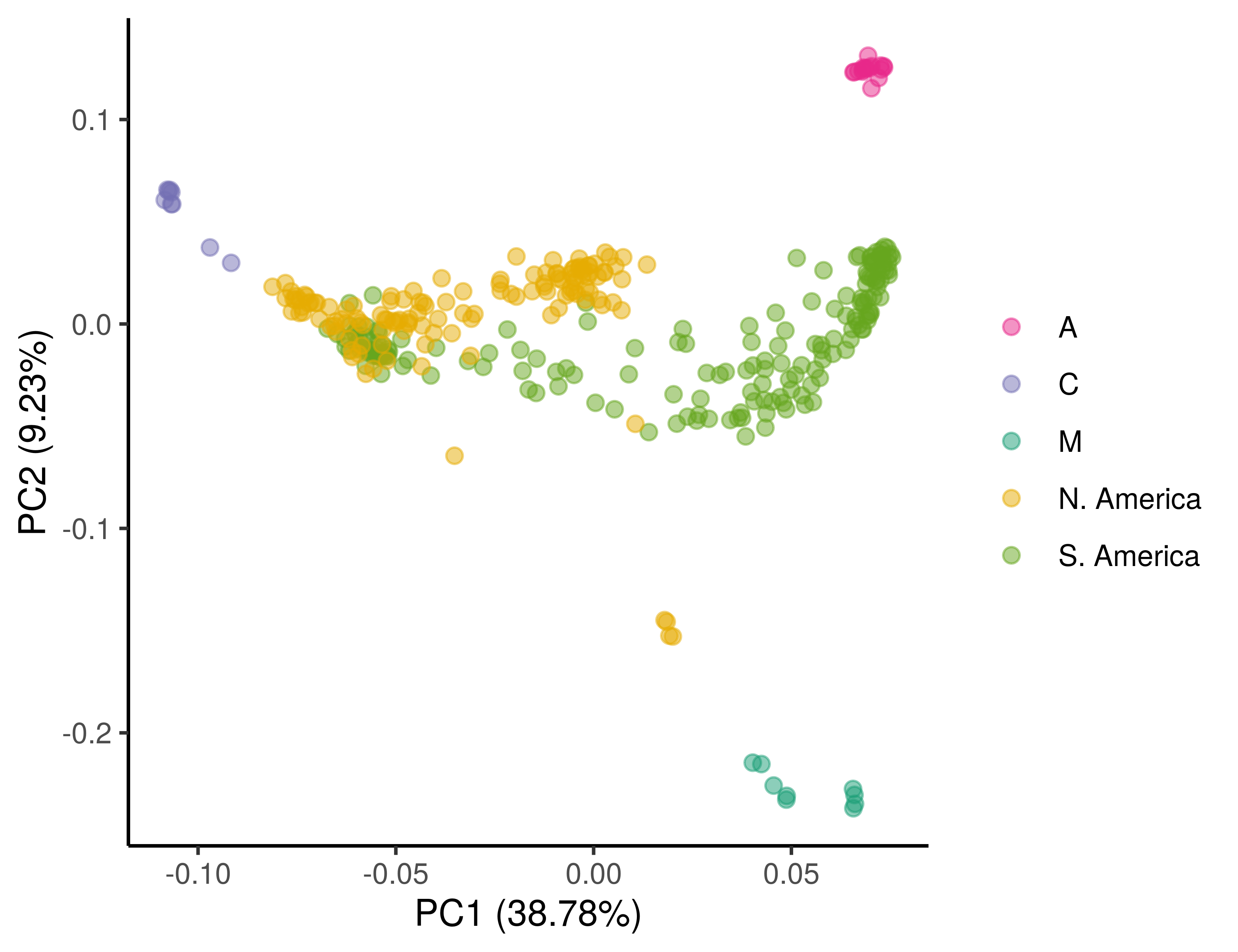

Supplement: S1 Fig — Principal components analysis generated in PCAngsd using genotype likelihoods from the same thinned set of 14,044 autosomal SNPs used in global admixture analysis. The major axes of diversity separate out C ancestry (PC1) and M ancestry (PC2). Consistent with 3-way admixture, all sampled bees from North and South America are intermediate on the PCA, in the triangle formed by reference panels for Apis mellifera scutellata from southern and eastern Africa (A), A. m. carnica from eastern Europe (C) and A. m. mellifera and A. m. iberiensis from western Europe (M). (TIF) [file pgen.1009038.s007.tif]

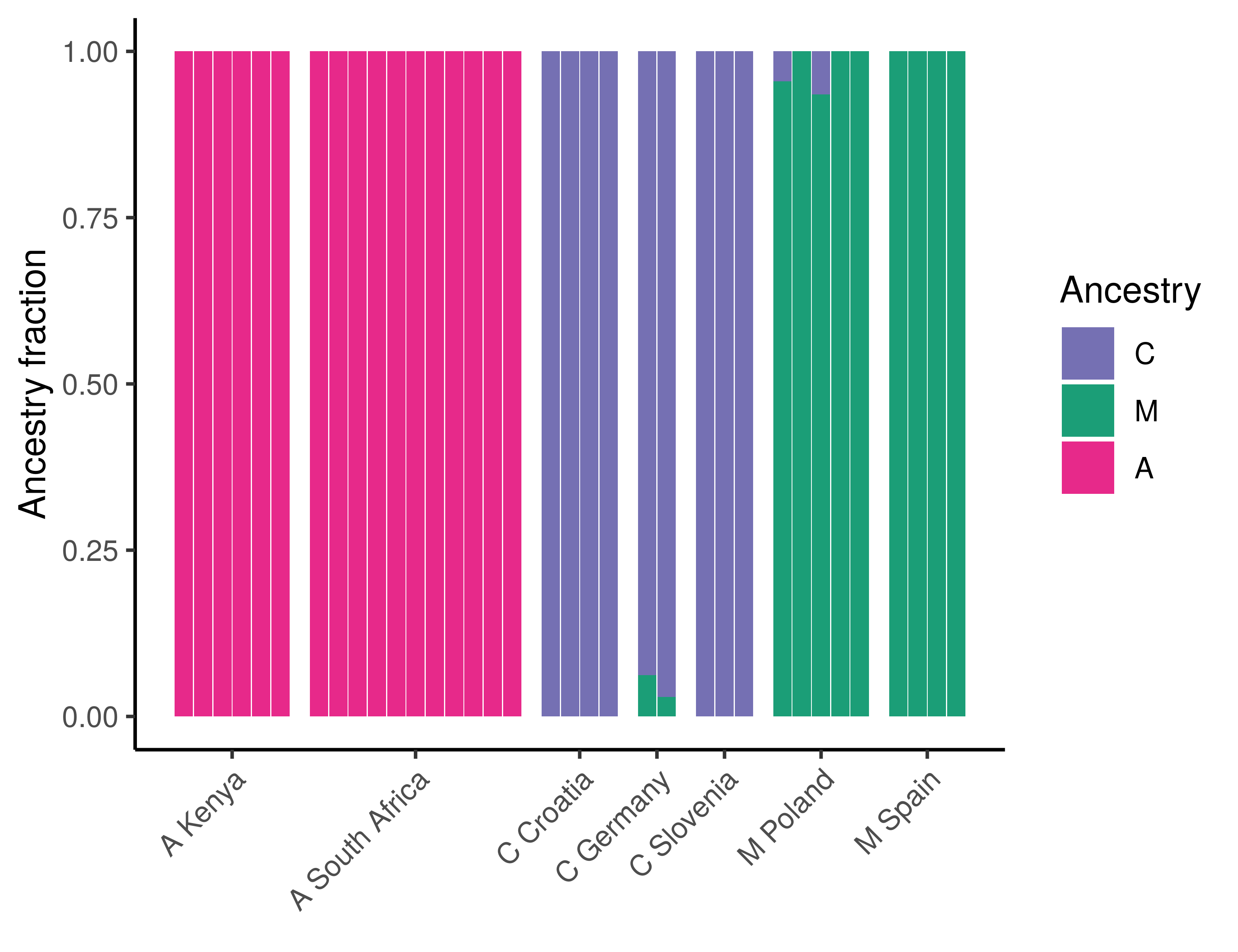

Supplement: S2 Fig — Results of NGSAdmix global admixture analysis for reference populations from the combined analysis of all populations (K = 3). These results were used to assign the unlabelled ancestry components output by NGSAdmix to A, C, and M groups, based on a clear mapping to the three reference populations. We see a small amount of admixture between C and M within our reference populations, which is consistent with limited gene flow from secondary contact within Europe. (TIF) [file pgen.1009038.s008.tif]

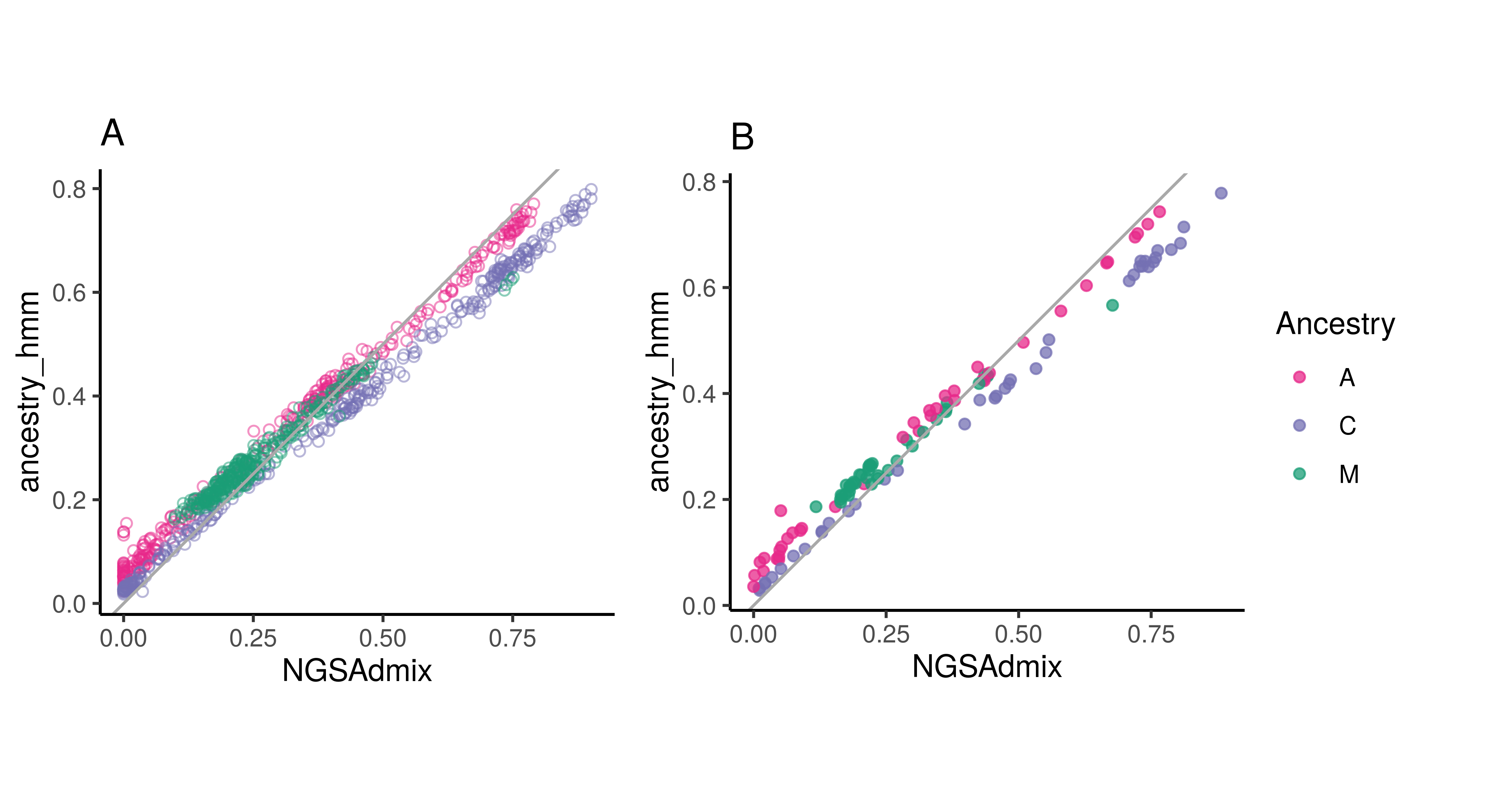

Supplement: S3 Fig — (A) Comparison of the mean genome-wide ancestry estimate from NGSAdmix (x-axis) and ancestry_hmm (y-axis) for each bee, with one-to-one line drawn in grey. The mean for the HMM is calculated by marginalizing the posterior over all ancestry states and taking a mean across SNPs. The individual-level ancestry estimates between the two methods agree strongly (Pearson’s correlation: 0.997 A, 0.999 C, 0.985 M), but the HMM estimates slightly higher minor ancestry for bees with low admixture proportions. (B) Population mean summarises for the same comparison of NGSAdmix vs. ancestry_hmm genome-wide ancestry estimates, with one-to-one line drawn in gray. Because the population mean ancestry proportions from NGSAdmix are used as a prior for the population-specific mixing proportions in ancestry_hmm, this panel can also be interpreted as the prior (x-axis) and posterior (y-axis) of the local ancestry HMM for population-level admixture proportions. (TIF) [file pgen.1009038.s009.tif]

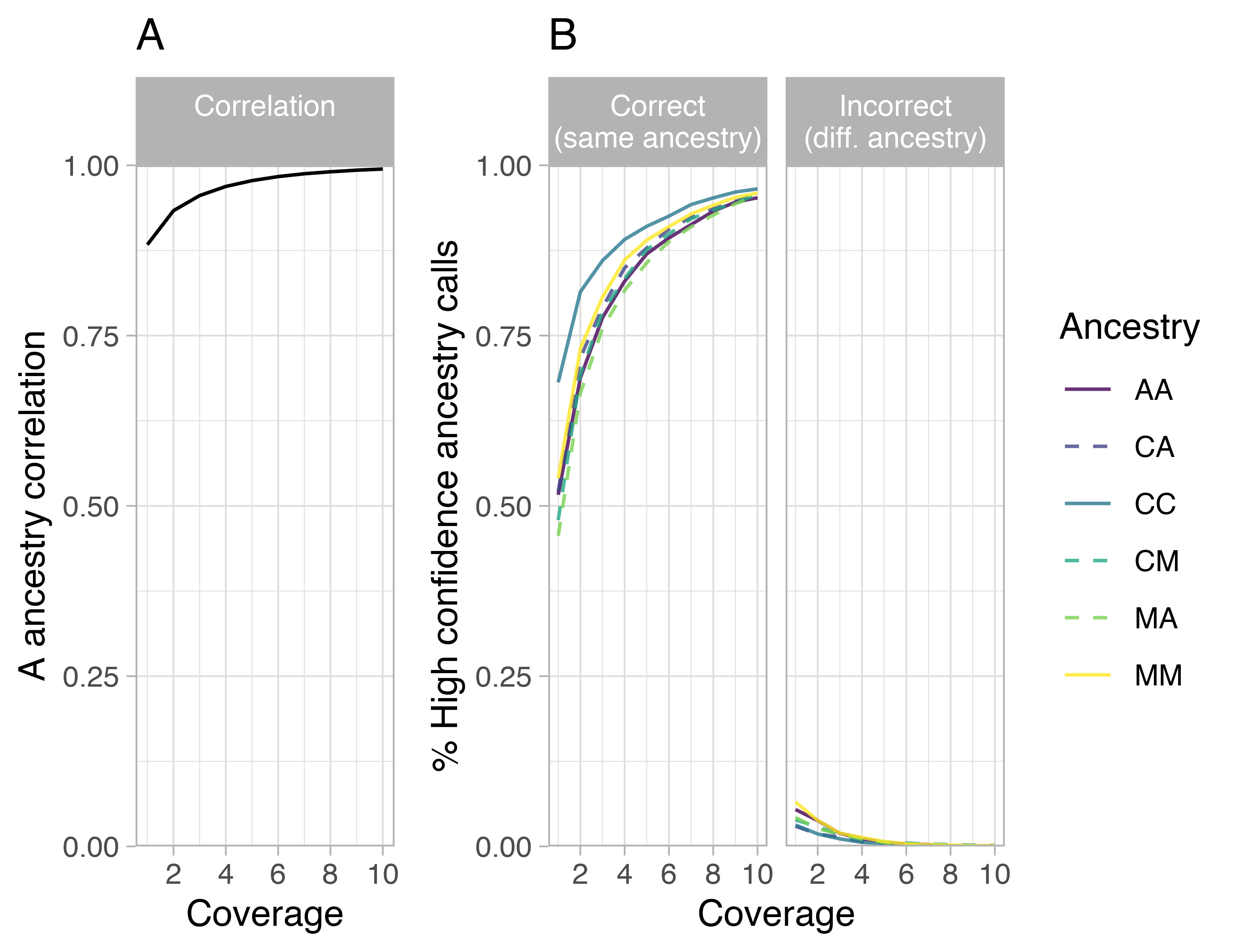

Supplement: S4 Fig — (A) Correlation between high-coverage and low-coverage ancestry calls, across different simulated depths of coverage (1-10x). (B) Proportion of high-confidence ancestry calls from high-coverage data that were replicated in analyses of low-coverage data, with different simulated depths of coverage (1-10x). These results are from a preliminary analysis of the power to call local ancestry accurately, used to inform target sequencing depth for this study. For this preliminary study, we used a published SNP set with data for A, C, and M reference populations [8] based on earlier versions of the honey bee genome (Amel4.5 [116]) and recombination map [33]. We enriched for ancestry-informativeness and thinned for linkage disequilibrium (≥ 0.2 MAF in at least one reference population and r2 < 0.4 within the A reference population), leaving 161k SNPs. First we ran ancestry_hmm [53] using called genotypes from a high-coverage admixed population with intermediate admixture proportions (Riverside 2014 (n = 8): 40% C, 20% M, 40% A ancestry). We simulated lower coverage data from this same population by generating a binomial sample of n reads for each locus, based on the individual’s genotype. To simulate realistic variance in coverage across the genome, n for each site and individual was generated from a negative binomial distribution with variance 3x the mean [117]. We additionally simulated a 1% sequencing error rate. Running local ancestry inference on the high coverage data, we inferred high confidence ancestry states for 81% of sites. First we calculated a point estimates for A ancestry (p(AA)+12(p(CA)+p(MA))) at every site for each individual and used these estimates to calculate a correlation between the high coverage ancestry calls and low coverage ancestry calls. Then we calculated the percent of high confidence calls that were replicated with high confidence (>0.8 posterior) in the low coverage data for the same ancestry state (“correct”) or a different ancestry state ( [file pgen.1009038.s010.tif]

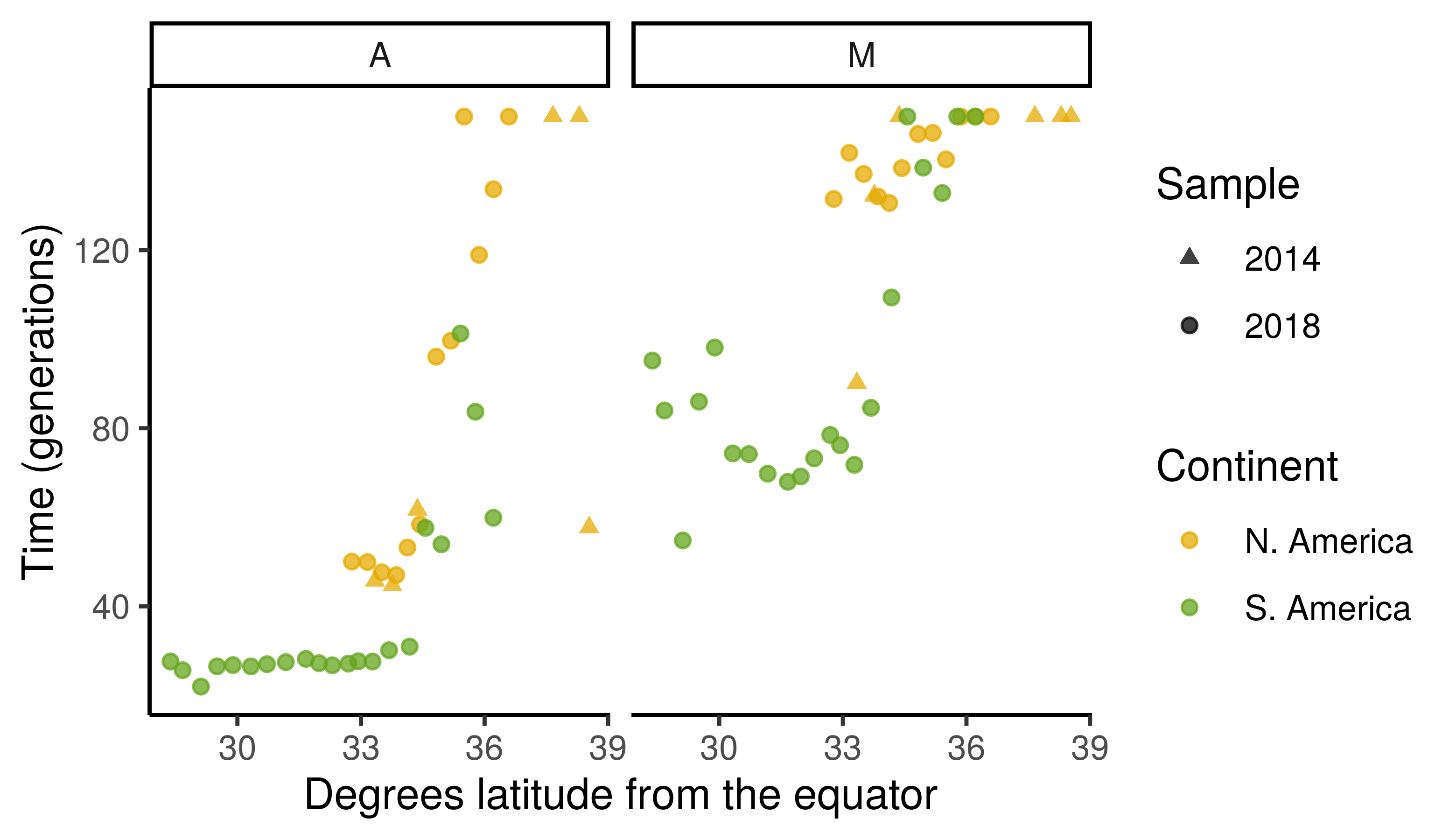

Supplement: S5 Fig — Inferred timing of migration pulses from A ancestry (left) and M ancestry (right). Each population’s admixture timing is estimated separately, during local ancestry inference (ancestry_hmm), and results are plotted across latitude. We allowed a range of 2-150 generations, so the highest time estimates are truncated at 150 generations. Admixture with scutellata (A) ancestry began in 1956, 62 years before sampling in 2018. We have little prior information about the timing of M into C admixture, which likely varies across the Americas, but in general should pre-date admixture with A. The number of generations per year for feral honey bee populations is uncertain. (TIF) [file pgen.1009038.s011.tif]

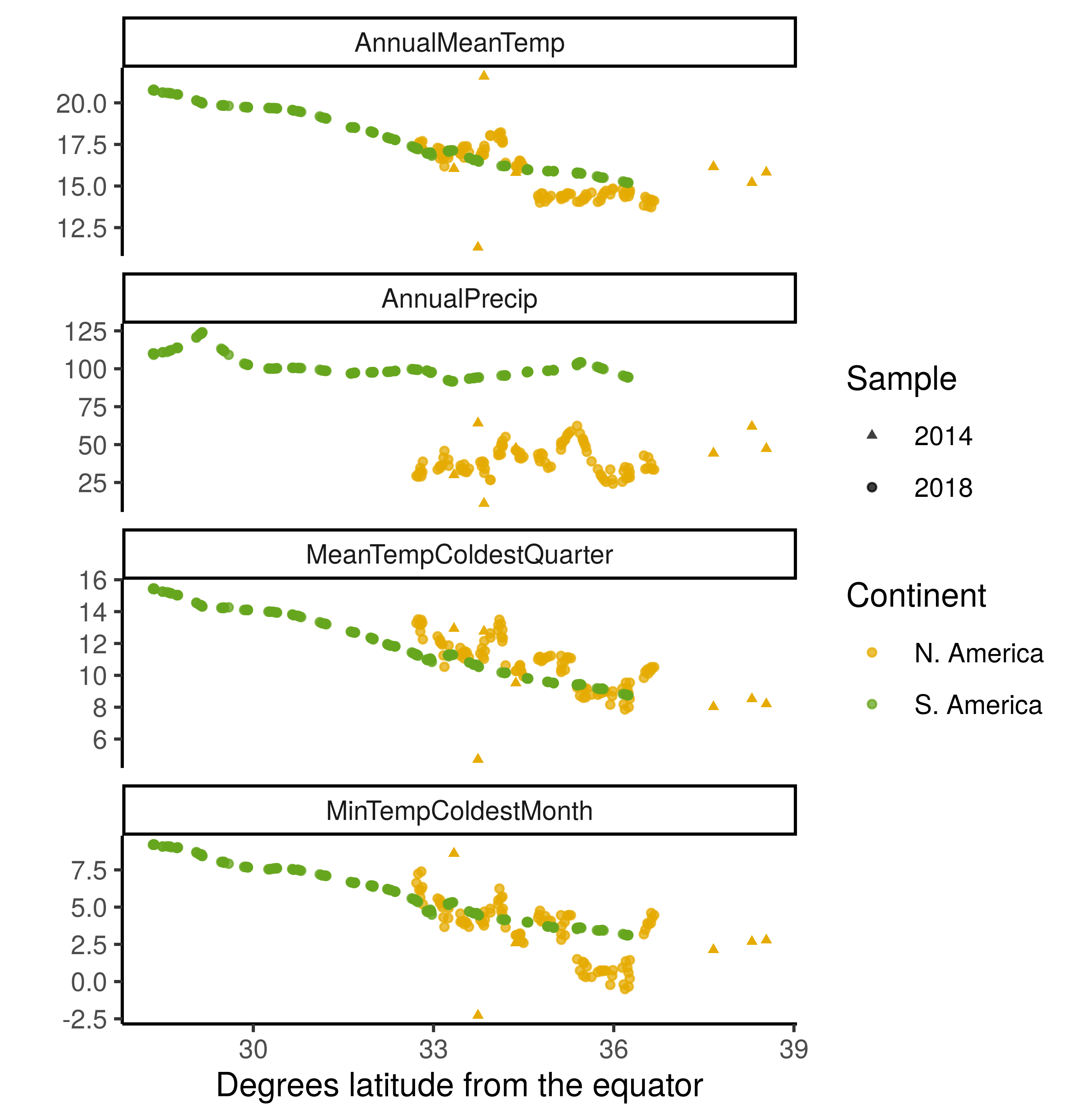

Supplement: S6 Fig — Bioclim climate variables for all sample sites plotted against latitude: (A) Mean annual temperature (B) Mean annual precipitation (C) Mean temperature coldest quarter (D) Minimum temperature coldest month. Two adjacent climate outliers in the N. American sample can be seen in the top two panels and represent bees from an inland desert (hot and dry) and a high altitude sampling site (cold and wet) at similar latitudes in Riverside County, CA. Bees from this same high altitude site are also outliers in the bottom two panels, having the coldest mean and minimum winter temperatures of all sites. (TIF) [file pgen.1009038.s012.tif]

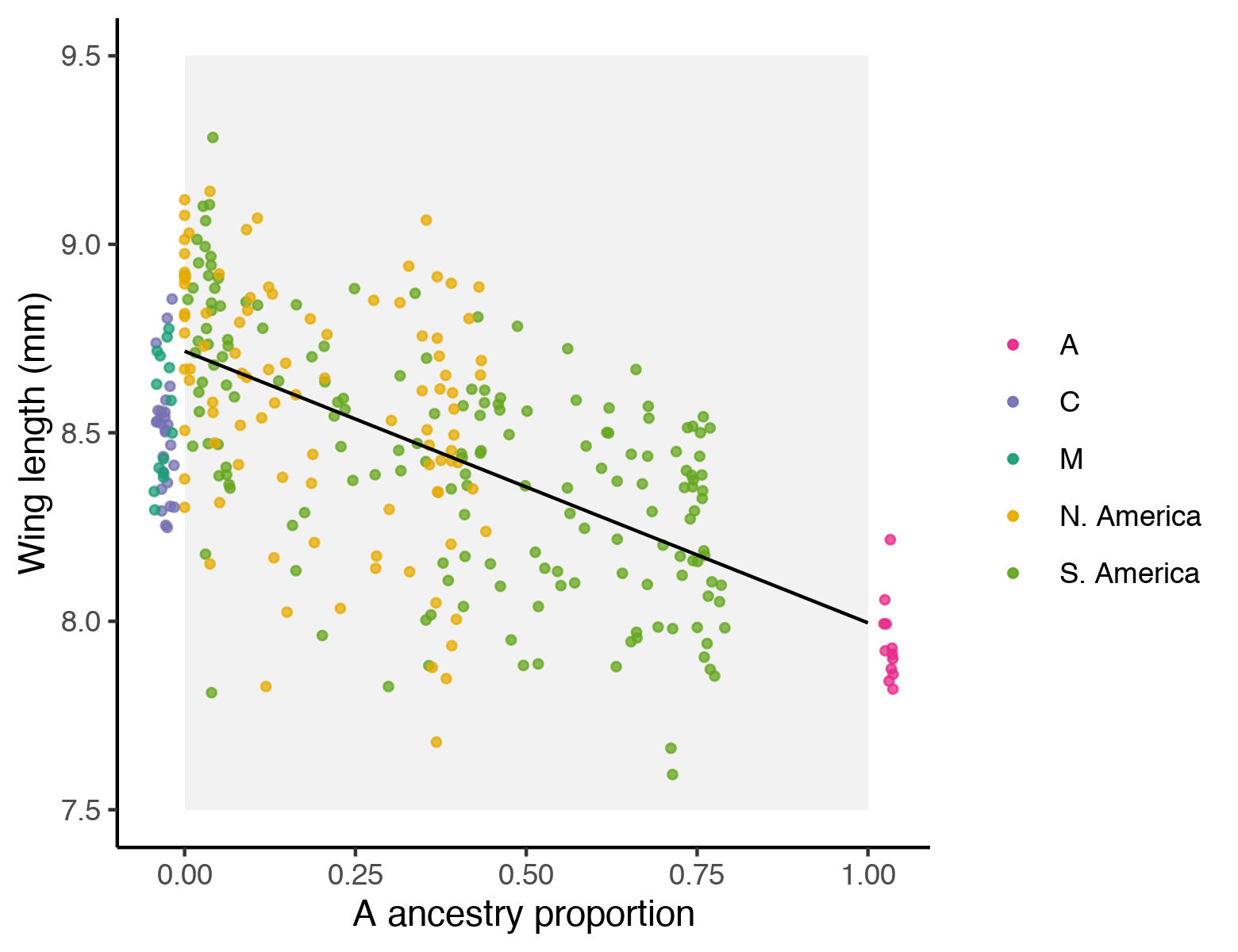

Supplement: S7 Fig — Individual honey bees are represented as points, with wing lengths plotted along the x-axis and genomewide A ancestry proportions (NGSAdmix results) along the y-axis. We draw the best-fit regression line (slope = -0.72 mm, F(1, 267) = 119, P = 3.65 × 10^−23^, R2 = 0.31, n = 269). We also include wing lengths for A, C and M reference bees from the Oberursel Collection, which we assume have none or full A ancestry. These reference bees are plotted slightly outside the range [0, 1] and with jitter to facilitate viewing individual points that would otherwise all cluster on the boundaries. (TIF) [file pgen.1009038.s013.tif]

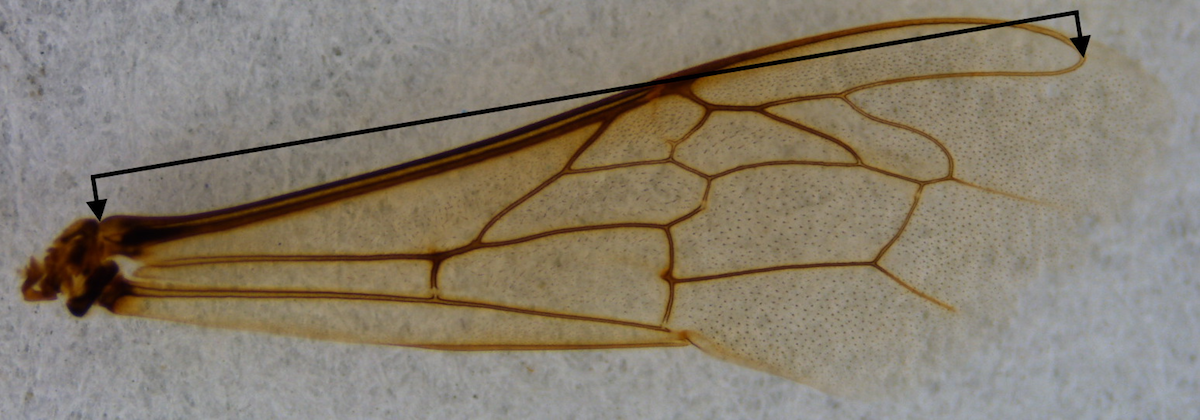

Supplement: S8 Fig — Fore wing image cropped and annotated to show length measurement taken. A full length to the tip of the wing is the standard measurement, but we use this alternative because many of our samples have significant wing tatter. (TIF) [file pgen.1009038.s014.tif]

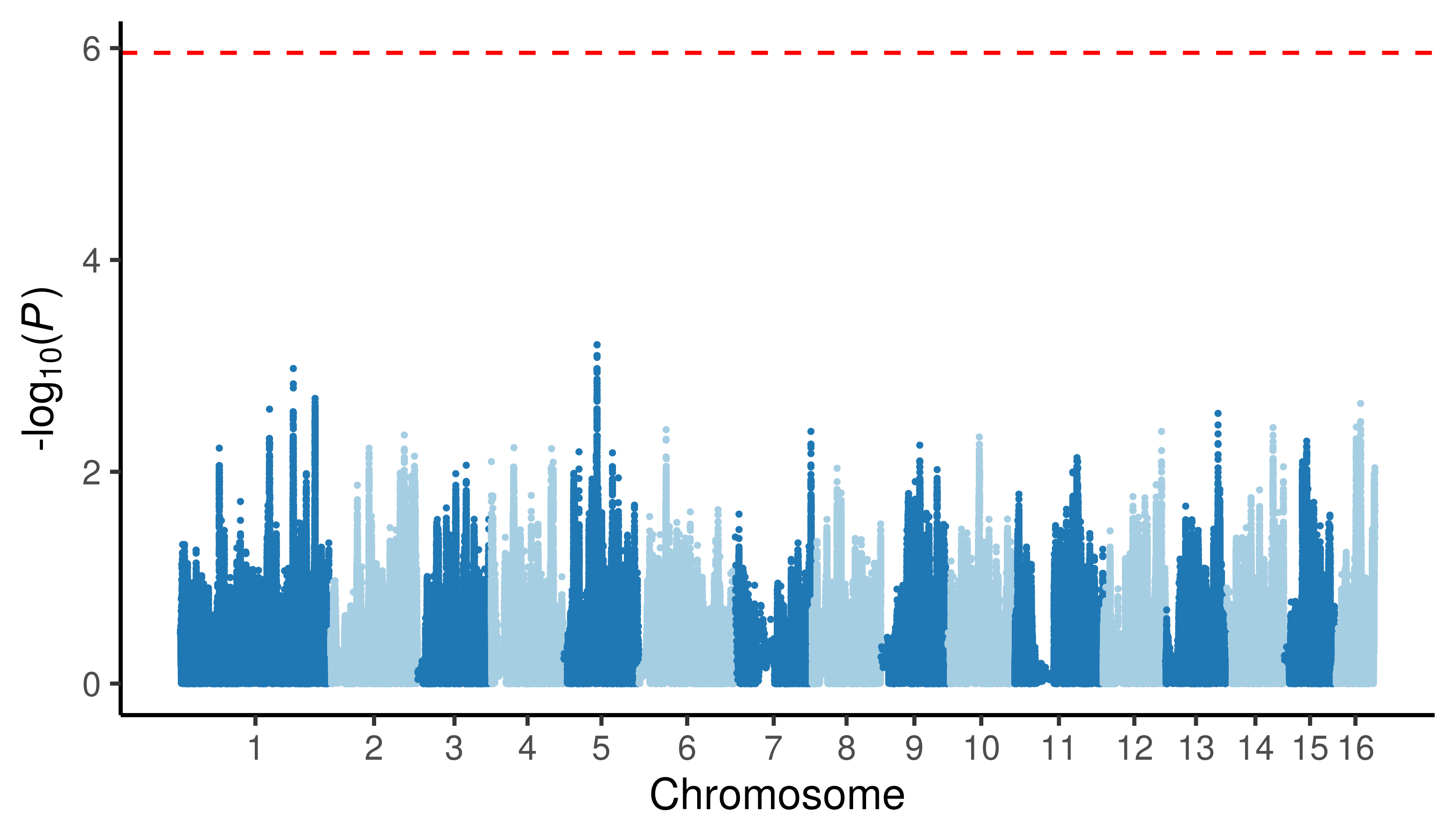

Supplement: S9 Fig — We plot the p-value for each SNP across the genome, based on independent tests of association between A ancestry at that SNP and wing length. The red dashed line marks the genome-wide significance threshold for a family-wise error rate of 0.05, using a two-tailed test. In admixture mapping, SNPs are correlated, and the number of independent statistical tests depends on the number of generations recombination has had to break up ancestry blocks. Here we use an analytical approximation for the significance threshold based on 47.65 generations of admixture (population median estimate). (TIF) [file pgen.1009038.s015.tif]

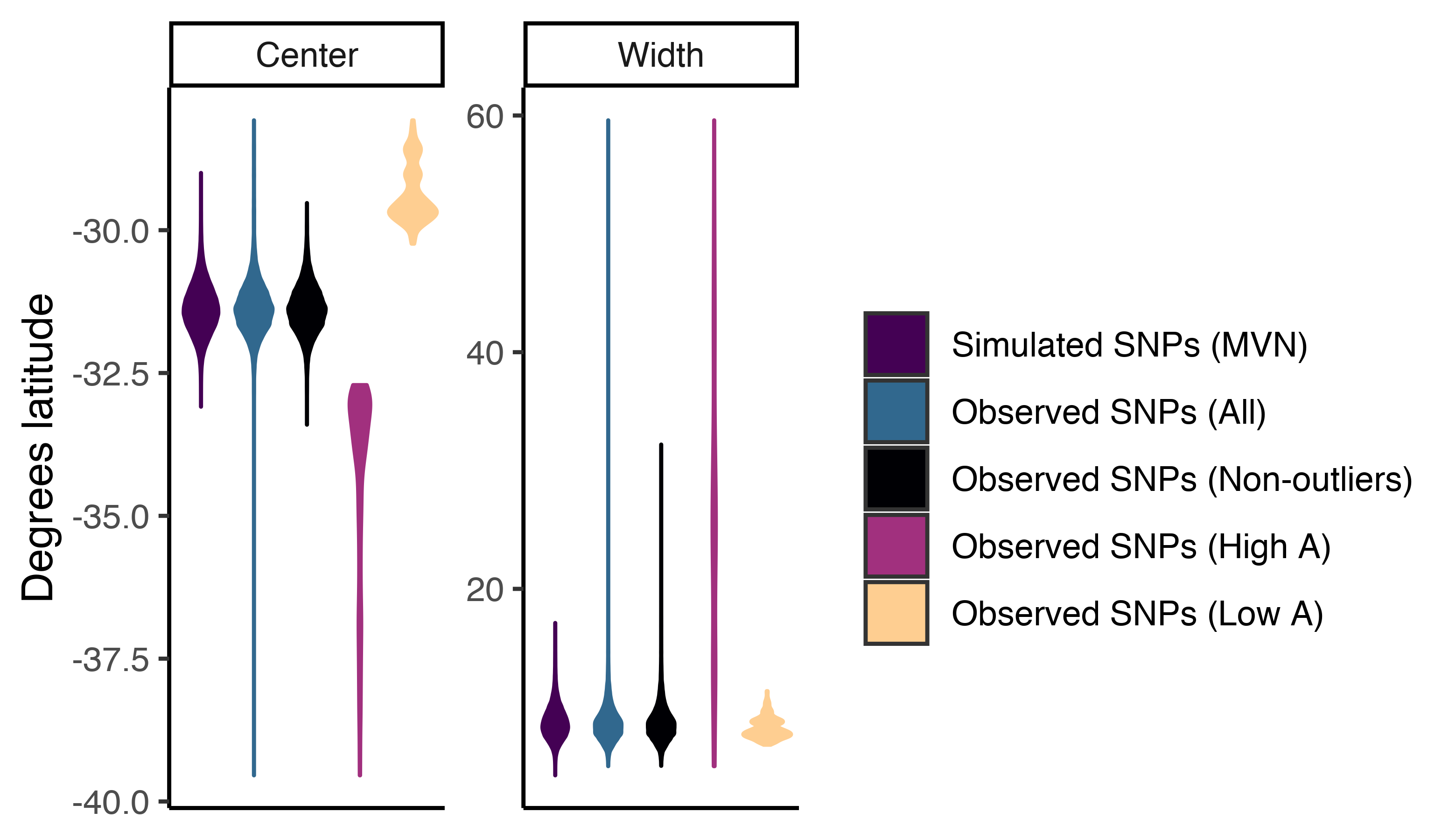

Supplement: S10 Fig — A logistic cline model was fit to observed and simulated population ancestry frequencies across latitude for S. America. Estimated cline parameters, center and width (w=|4b|), are presented as violin plots. Units for both cline center and width are degrees latitude. We additionally partition observed SNPs by outlier and non-outlier status, set by 10% FDR for high or low A ancestry in South America. Individual SNP clines were only fit in South America, where we observed the full cline. (TIF) [file pgen.1009038.s016.tif]

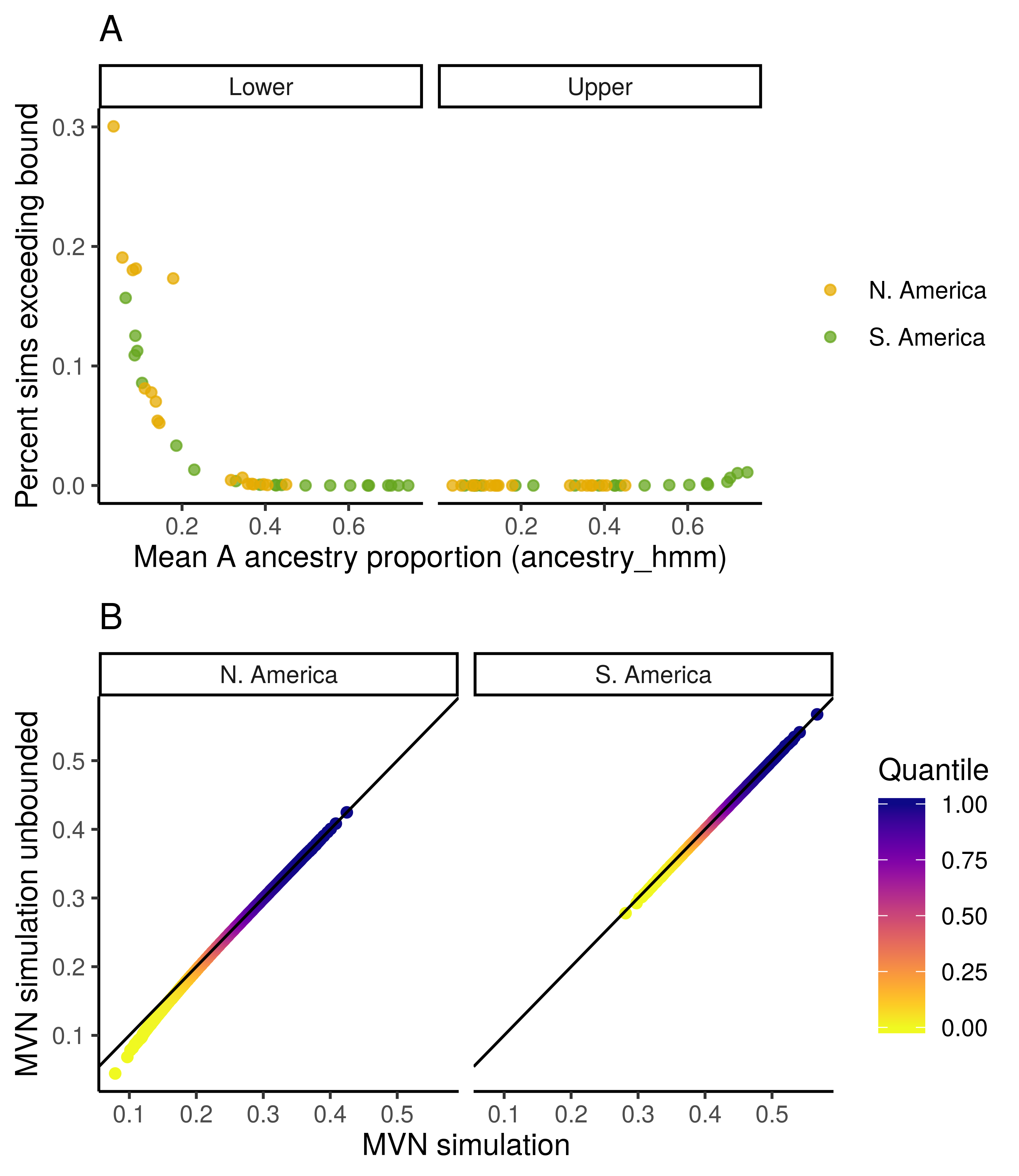

Supplement: S11 Fig — (A) Percent of simulated population A-ancestry frequencies exceeding lower (left) and upper (right) bounds, and thus truncated to [0, 1] range. Each population is a point and the populations most affected by truncation are low-A ancestry populations with mean A-ancestry proportions close to the bound at 0. (B) QQ-plot comparing the quantiles for mean A ancestry before and after truncation in N. America (left) and S. America (right). The distribution of mean A ancestry is mostly unaffected by restricting simulated population A ancestry frequencies to the [0, 1] range, but truncation does reduce model predictions of very low A-ancestry frequencies in N. America, where mean A ancestry is already low. (TIF) [file pgen.1009038.s017.tif]

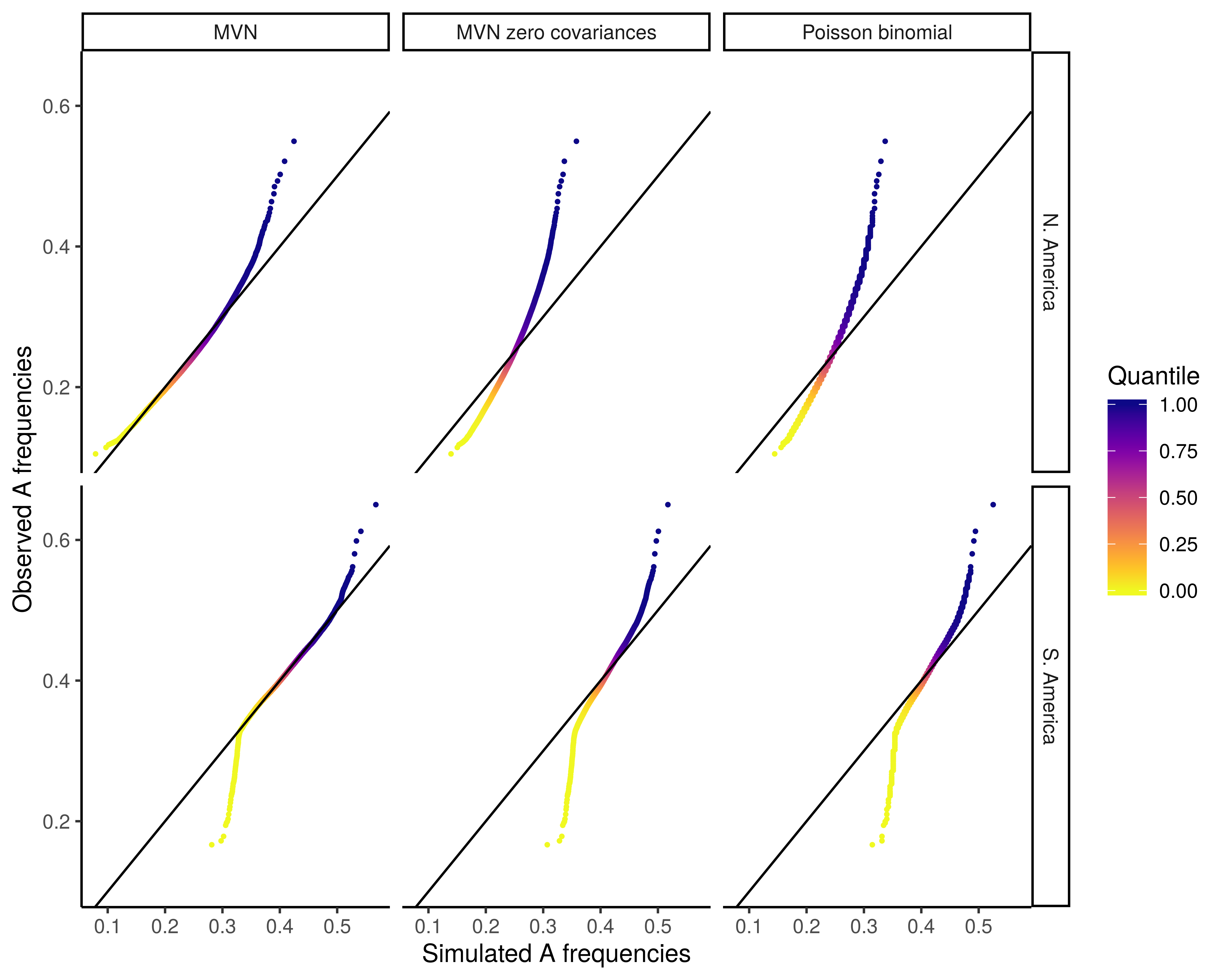

Supplement: S12 Fig — QQ-plots comparing observed quantiles for mean A ancestry in North America (top) and South America (bottom) to the quantiles generated by three simulated distributions (left-to-right): MVN, MVN with zero covariances, and Poisson binomial model. Only the MVN model, allowing for covariances between populations, matches the bulk of the observed distribution. (TIF) [file pgen.1009038.s018.tif]

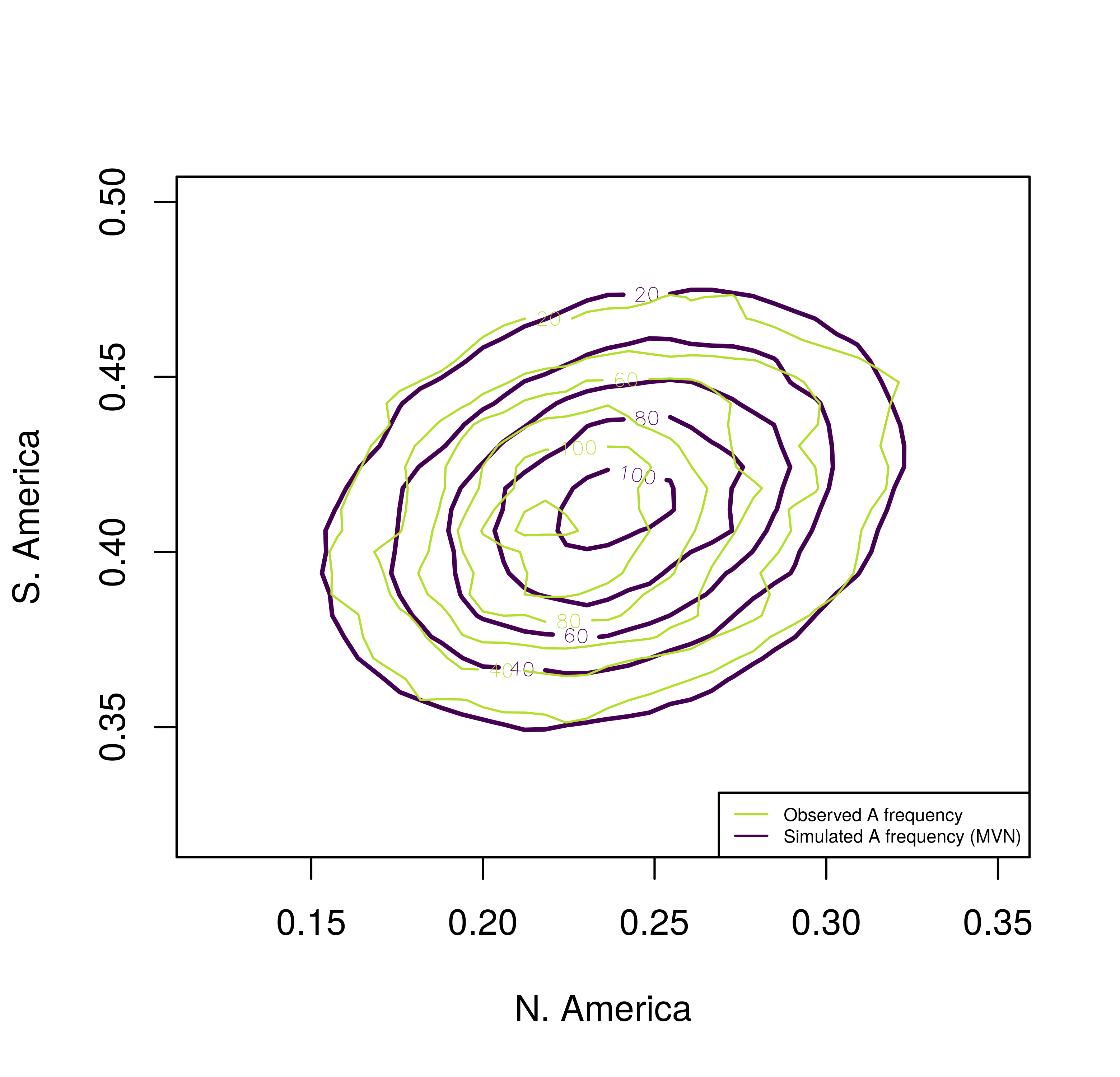

Supplement: S13 Fig — (TIF) [file pgen.1009038.s019.tif]

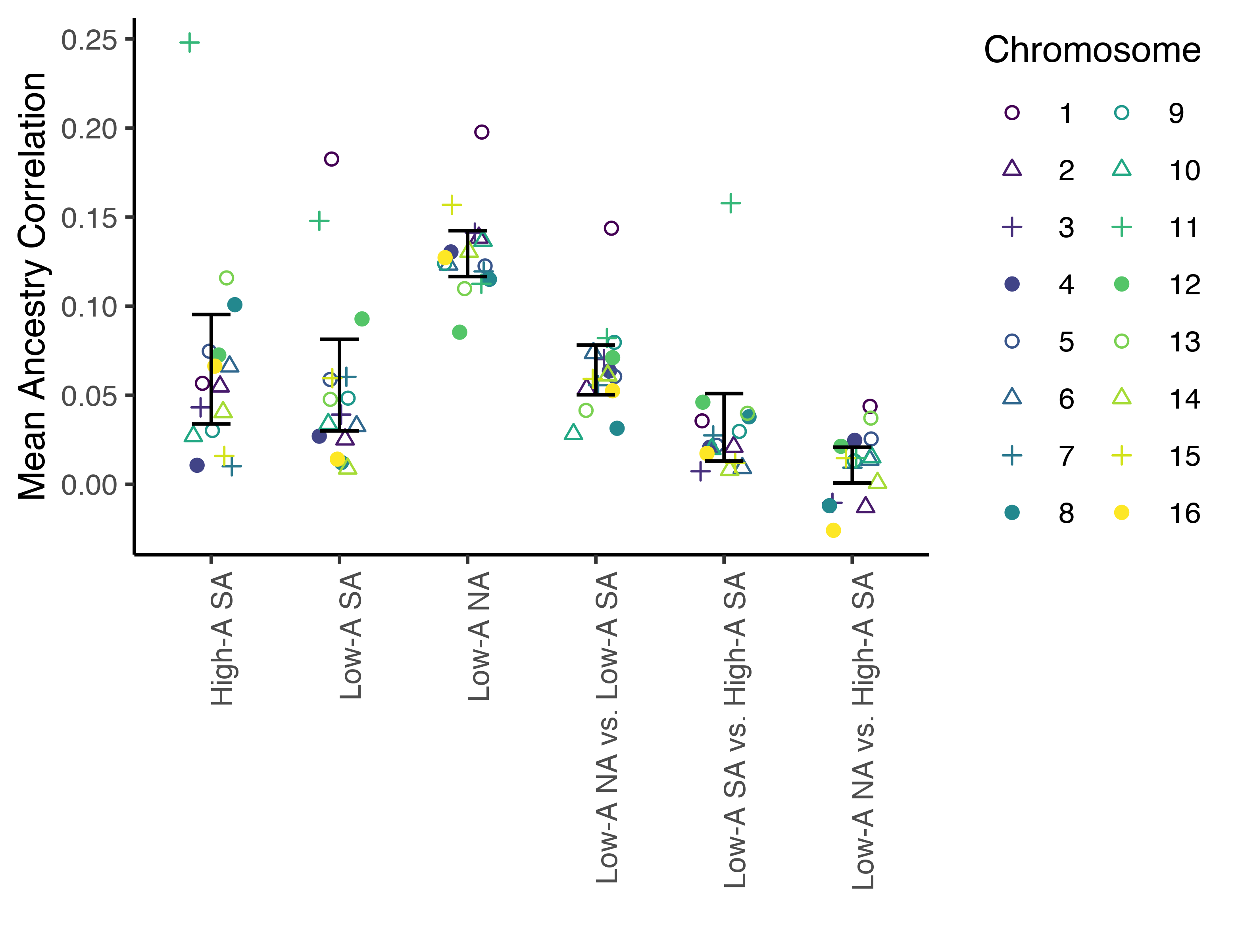

Supplement: S14 Fig — Mean ancestry covariances (K matrices) were calculated separately for each chromosome, using the genome-wide mean ancestry as α, then correlations were summarised by taking the mean for each type of population comparison, within and between continents and low vs. high A regions. Error bars show the normal-approximated 95% confidence intervals around these means. We divided populations in South America into two groups , ‘low A’ and ‘high A’, relative to the cline center. Low A populations are found at higher latitudes and correspondingly cooler climates. All North American samples come from the low-A side of the cline. On average across chromosomes, low-A South American populations share higher ancestry correlations with low-A North American populations than with geographically closer high-A South American populations (0.032: CI95[0.011,.053], P = .005, paired 2-sided t-test). Two chromosomes harbor large outlier regions consistent with their elevated correlations shown here: Chromosome 1 has a large cluster of loci with high A ancestry in North and South America while chromosome 11 has a wide region of low A ancestry exclusive to South America. The results do not change qualitatively if these two outlier chromosomes are both removed from the analysis (0.035: CI95[0.022,0.047], P = 4.8x10^−5^, paired 2-sided t-test). (TIF) [file pgen.1009038.s020.tif]

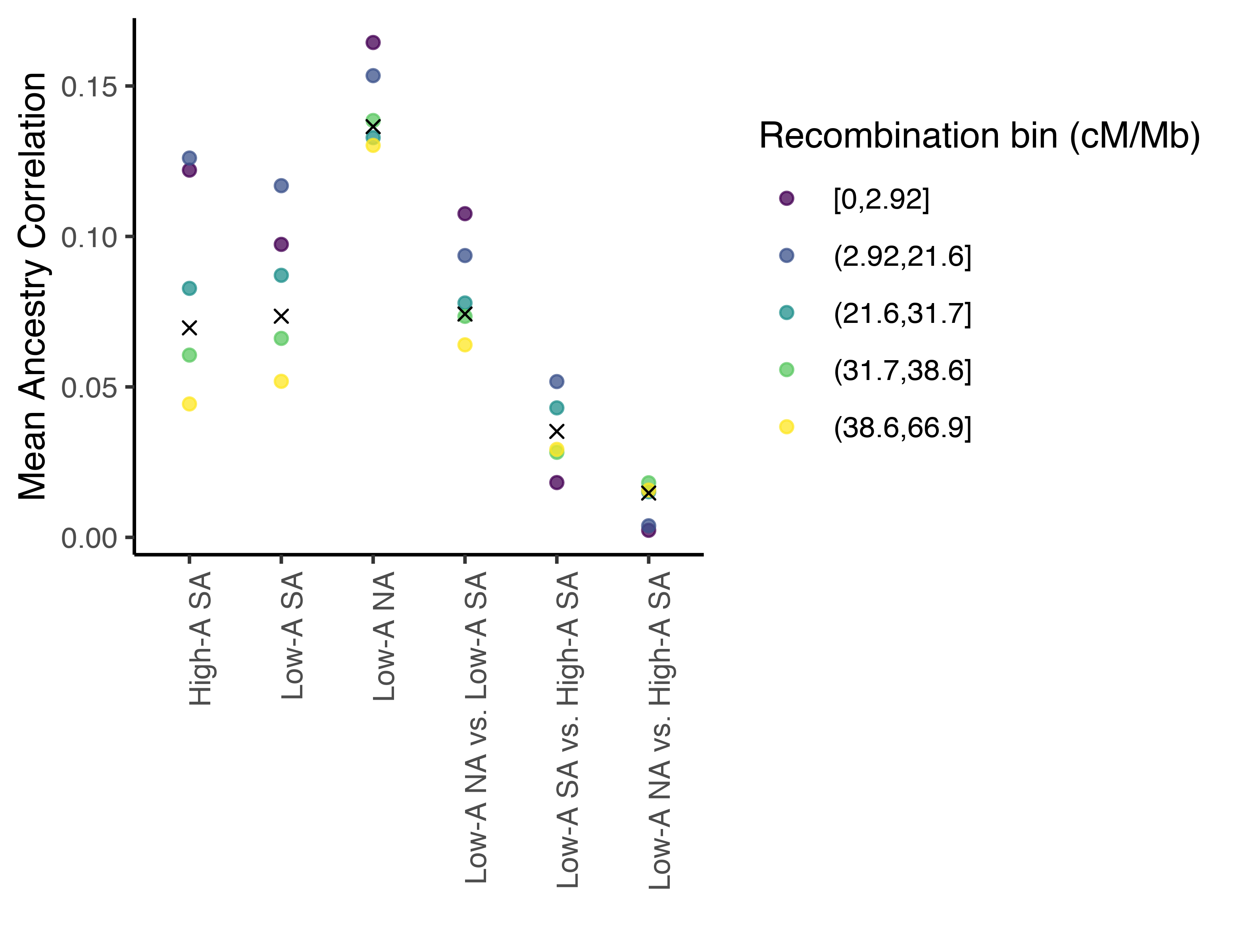

Supplement: S15 Fig — Mean ancestry covariances (K matrices) were calculated separately for each of the 5 recombination rate quintiles, using the genome-wide mean ancestry as α, then correlations were summarised by taking the mean for each type of population comparison, within and between continents and low vs. high A regions. About half of the South American populations, and all of the sampled North American populations come from the low-A side of the hybrid zone (relative to the estimated cline center). The genomewide mean is additionally shown as an X. On average across recombination bins, low-A South American populations share higher ancestry correlations with low-A North American populations than with geographically closer high-A South American populations (0.049: CI95[0.021,.078], P = .009, paired 2-sided t-test). (TIF) [file pgen.1009038.s021.tif]

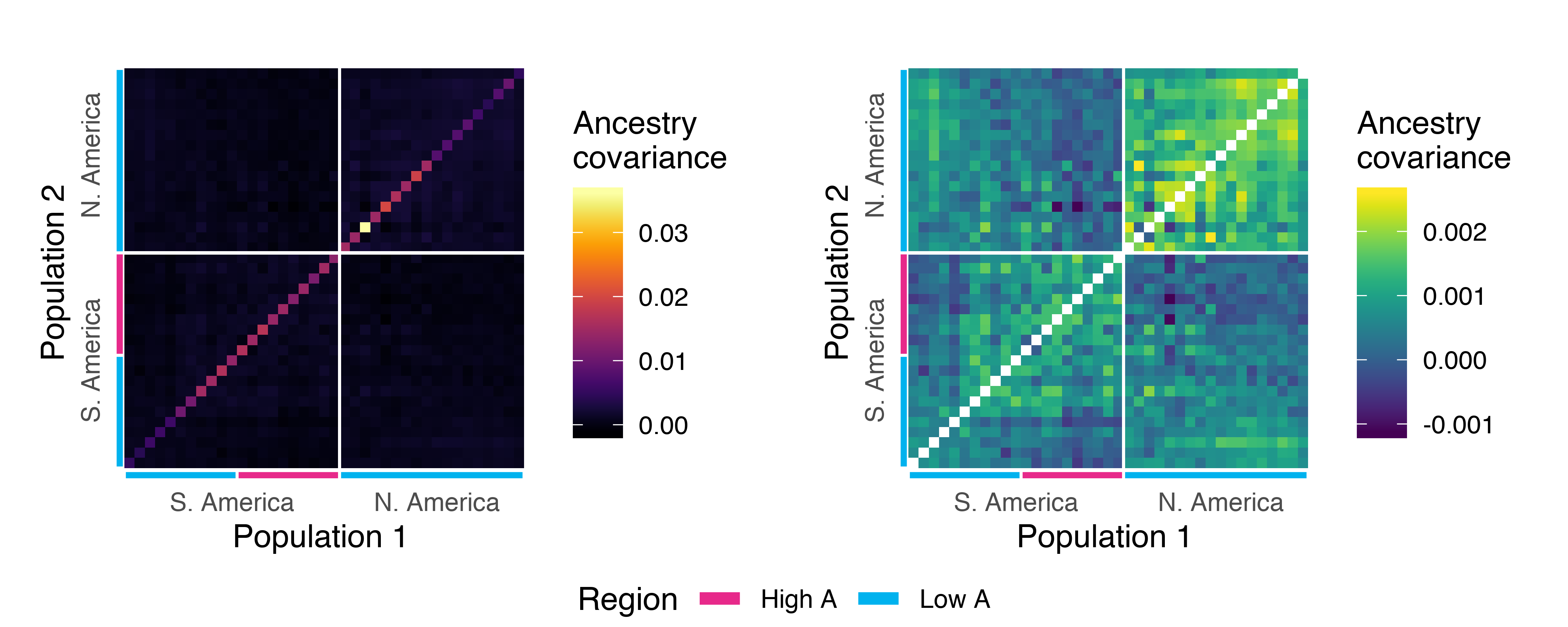

Supplement: S16 Fig — Ancestry covariance matrix (see Methods). Populations are ordered by latitude, with high and low A sides of each hybrid zone defined relative to the estimated genomewide cline center. Within-population variances (left) are shown separately from between-population covariances (right) because of the drastically different scales. Populations near the cline center have higher ancestry variances (most clearly seen in the diagonal elements) because they have A ancestry proportions closer to 50%. Drift and finite sample sizes also contribute to the observed ancestry variances. The third lowest latitude population in the North American cline with exceptionally high ancestry variance is Avalon, sampled from Catalina Island off the coast of California. The observed excess covariance between the two distant ends of these clines is unexpected under a simple model of spread North and South out of Brazil. (TIF) [file pgen.1009038.s022.tif]

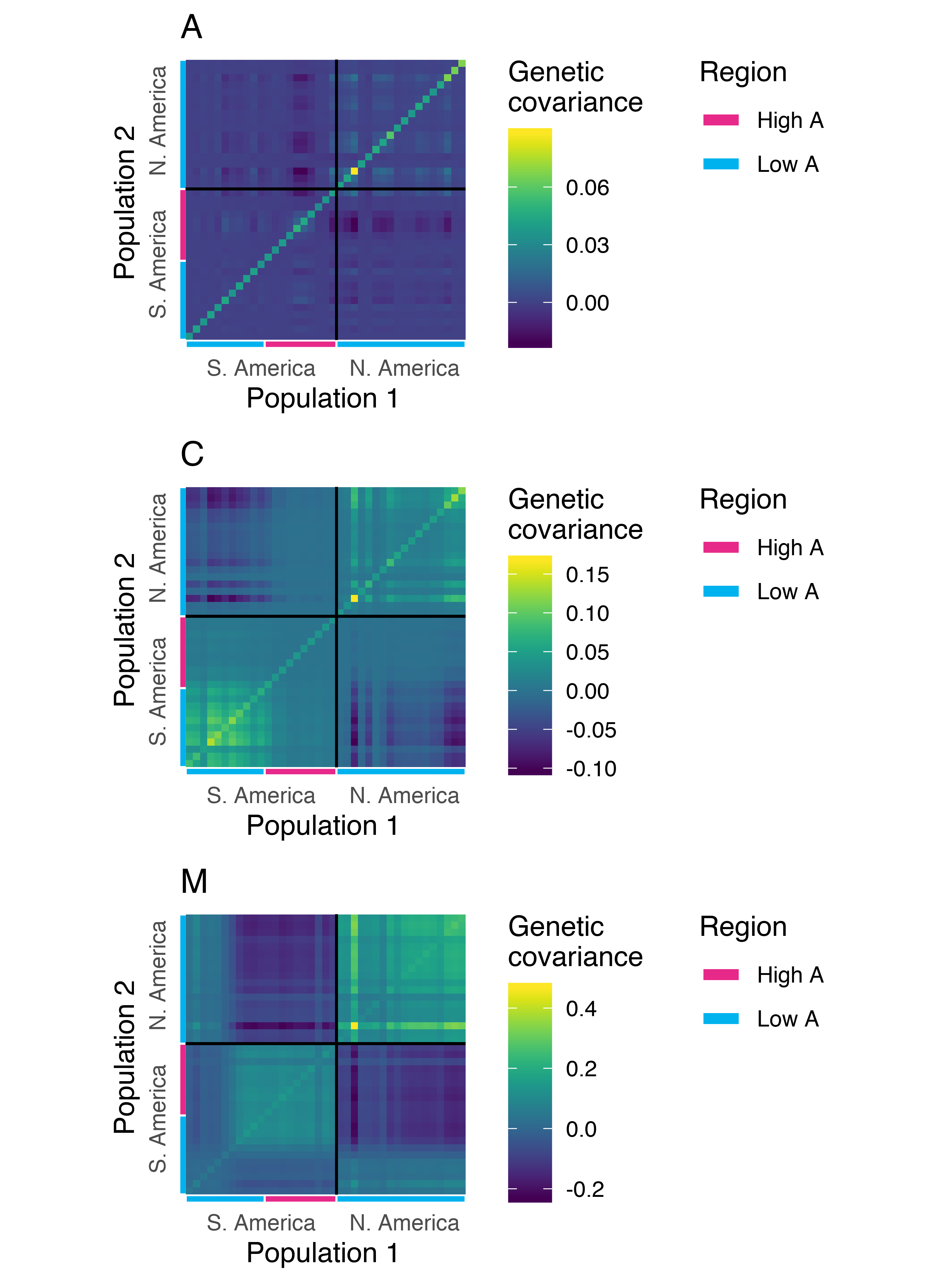

Supplement: S17 Fig — Genetic covariances within A (top), C (middle) and M (bottom) ancestry. Colors represent the population mean genetic covariance between individuals, and the range of values varies by ancestry (note: color bars have different scales). While kinship creates strictly positive covariances, here we observe some negative values because we can only calculate co-variation around the empirical mean combined sample allele frequency, not the true ancestral allele frequency (which is unknown). Population mean covariances were summarised from an individual-by-individual covariance matrix generated using PCAngsd from bam files filtered to only include regions of the genome with high confidence homozygous ancestry calls for the focal ancestry (posterior >0.8 from ancestry_hmm). High and low A sides of each hybrid zone are defined relative to the estimated genomewide cline center. (TIF) [file pgen.1009038.s023.tif]

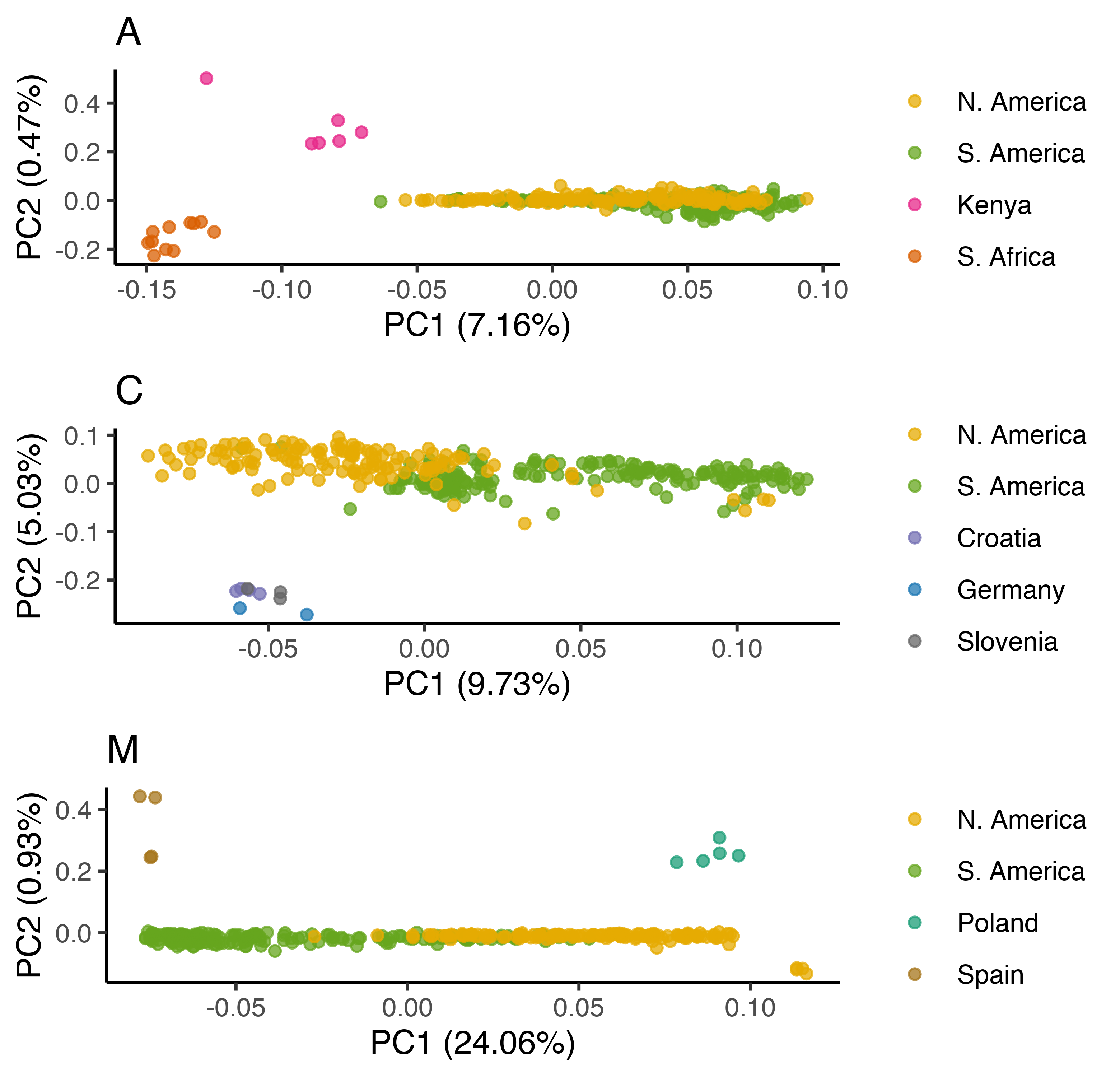

Supplement: S18 Fig — PCA analysis of A (top), C (middle) and M (bottom) ancestry. Analysis was performed using PCAngsd using all reference samples of the focal ancestry and sequence data from the hybrid zones filtered to only include regions of the genome with high confidence homozygous ancestry calls for the focal ancestry (posterior >0.8 from ancestry_hmm). Each bee is a point, colored by sample location. The two hybrid zones form somewhat separable clusters for European (C and M) ancestry, but not scutellata (A) ancestry. The major axis of genetic diversity within M ancestry in the Americas (PC1) mirrors pre-existing population structure within Europe between Apis mellifera mellifera (Poland) and Apis mellifera iberiensis (Spain), two well-known honeybee subspecies that may have different historical import rates to different regions. (TIF) [file pgen.1009038.s024.tif]

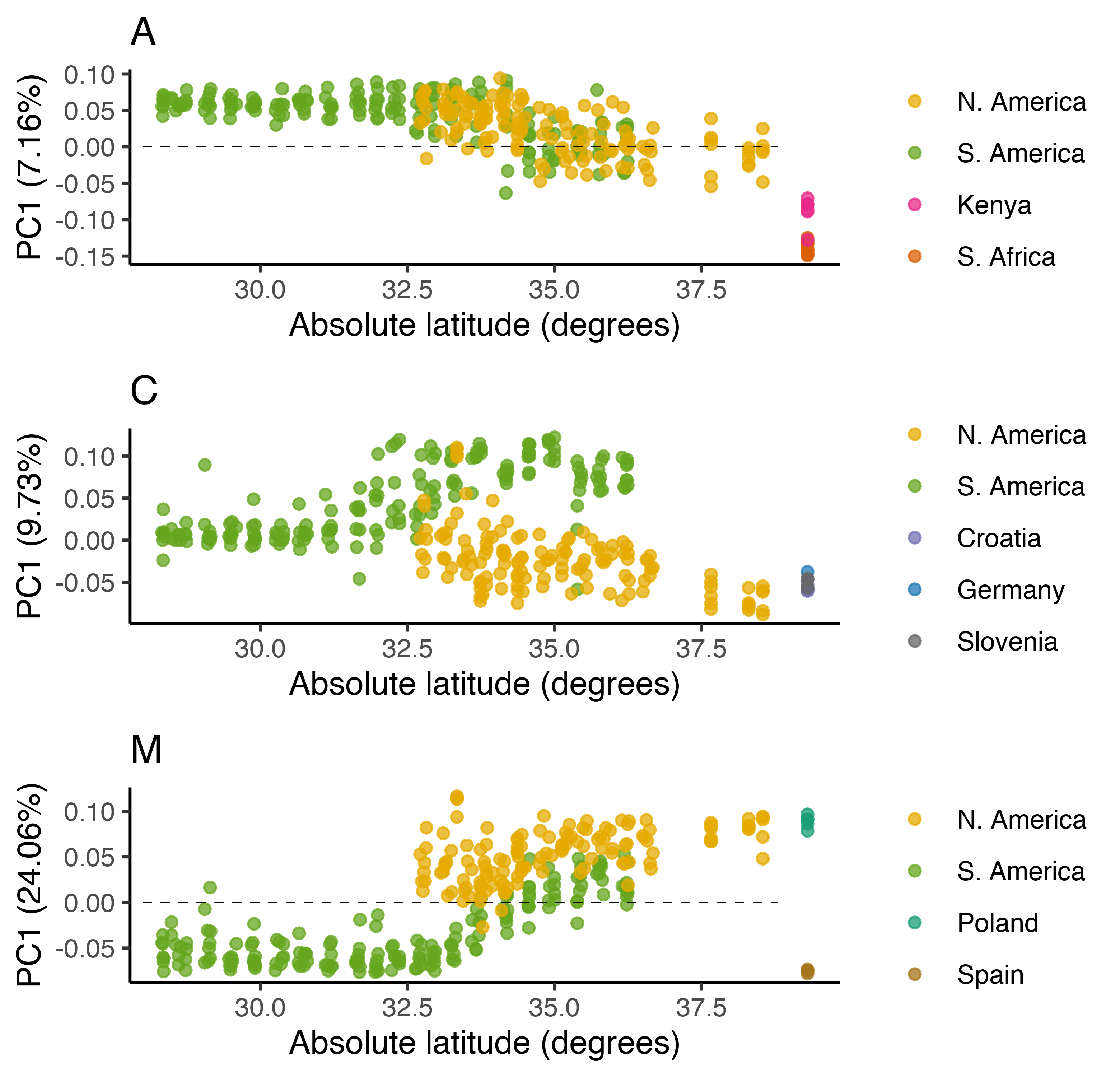

Supplement: S19 Fig — Here we plot the first principal component for genetic diversity within A (top), C (middle) and M (bottom) ancestry against absolute latitude of sampling location within the hybrid zone (see S18 Fig for original PCA). Each bee is a point and reference bees are plotted to the side (not at their actual latitude). Bees are colored by sample location. Note that bees with very low amounts of the focal ancestry (higher latitudes A ancestry or lower latitudes C ancestry) fall close to zero on PC1 (dashed line), which, despite using a method designed to account for low coverage data (PCAngsd), may simply be an artifact of low information per individual bee for genetic diversity within a low-frequency ancestry. A ancestry shows very little population structure along PC1 by continent or latitude. C is the dominant ancestry at higher latitudes in both zones and shows greater separation between the two ends of the zones (higher absolute latitude for both) than within South America. M ancestry at lower latitudes in South America is more similar to Apis mellifera iberiensis (Spain) than M ancestry elsewhere in the Americas. (TIF) [file pgen.1009038.s025.tif]

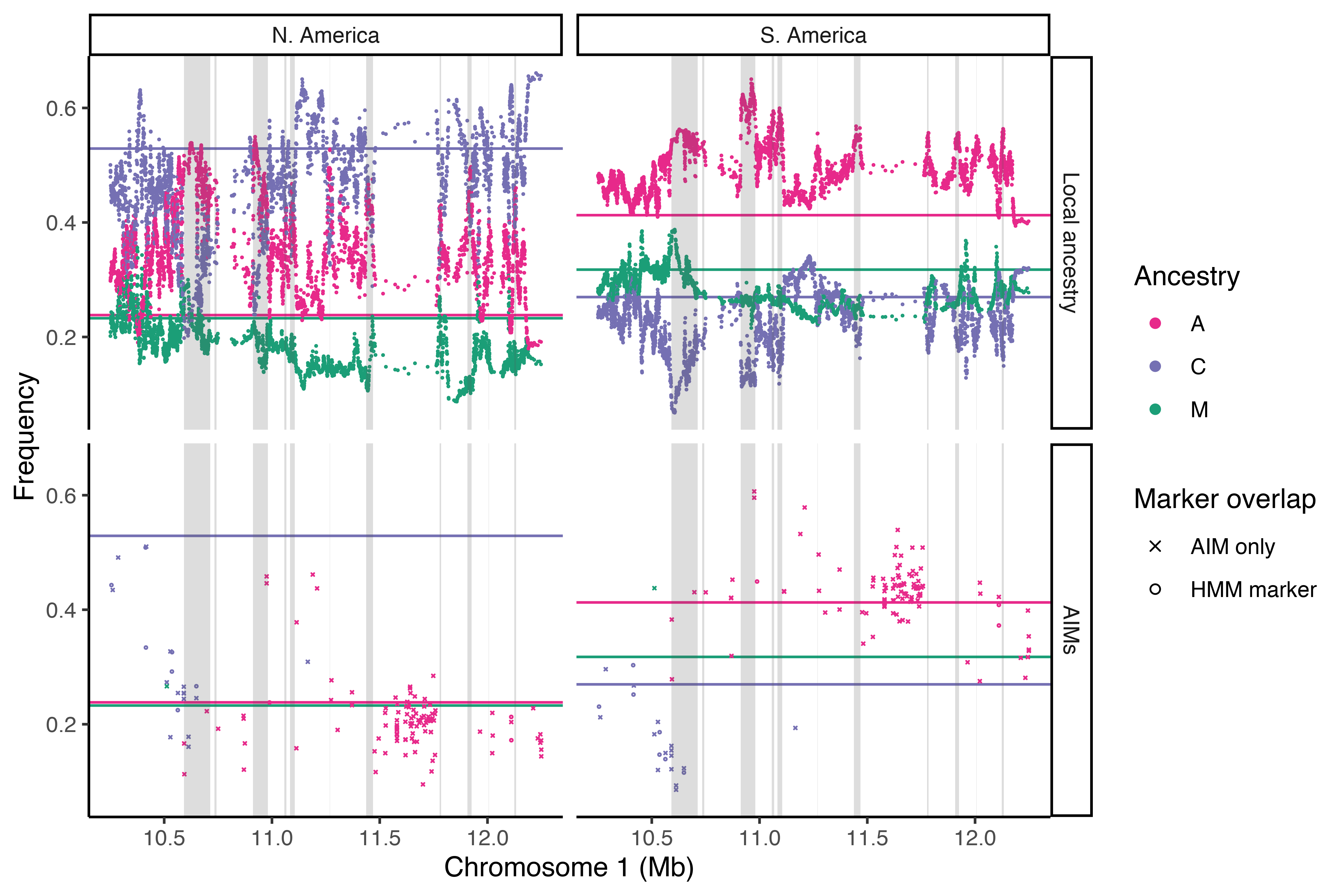

Supplement: S20 Fig — Zoomed-in view of the region on chromosome 1 with a cluster of high A ancestry peaks in both North America (left) and South America (right), with shared outlier regions meeting a 10% FDR for high A ancestry on both continents shaded in grey. (Top) Scutellata (A), western European (M) and eastern European (C) local ancestry estimates at each HMM marker. (Bottom) Mean frequency of ancestry informative markers AIMs (see Methods), most of which were not included in the ancestry_hmm inference (‘AIM only’) due to thinning. (TIF) [file pgen.1009038.s026.tif]

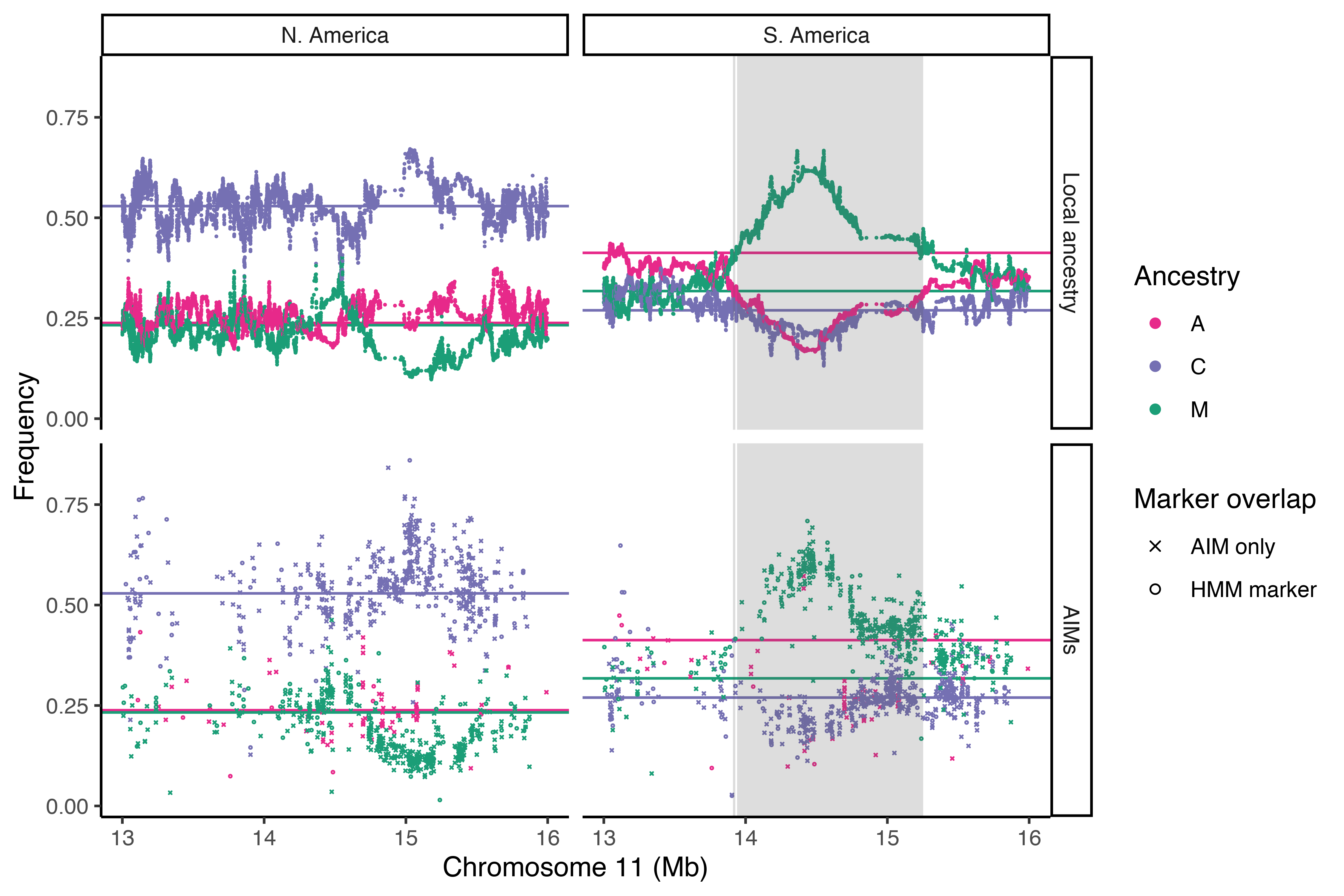

Supplement: S21 Fig — Zoomed-in view of the 1.4Mb region on chromosome 11 with high western European ancestry (M) in South America (right) but not in North America (left), with the outlier region meeting 10% FDR highlighted in grey. (Top) Scutellata (A), western European (M) and eastern European (C) local ancestry estimates at each HMM marker. (Bottom) Mean frequency of ancestry informative markers AIMs (see Methods), most of which were not included in the ancestry_hmm inference (‘AIM only’) due to thinning. (TIF) [file pgen.1009038.s027.tif]

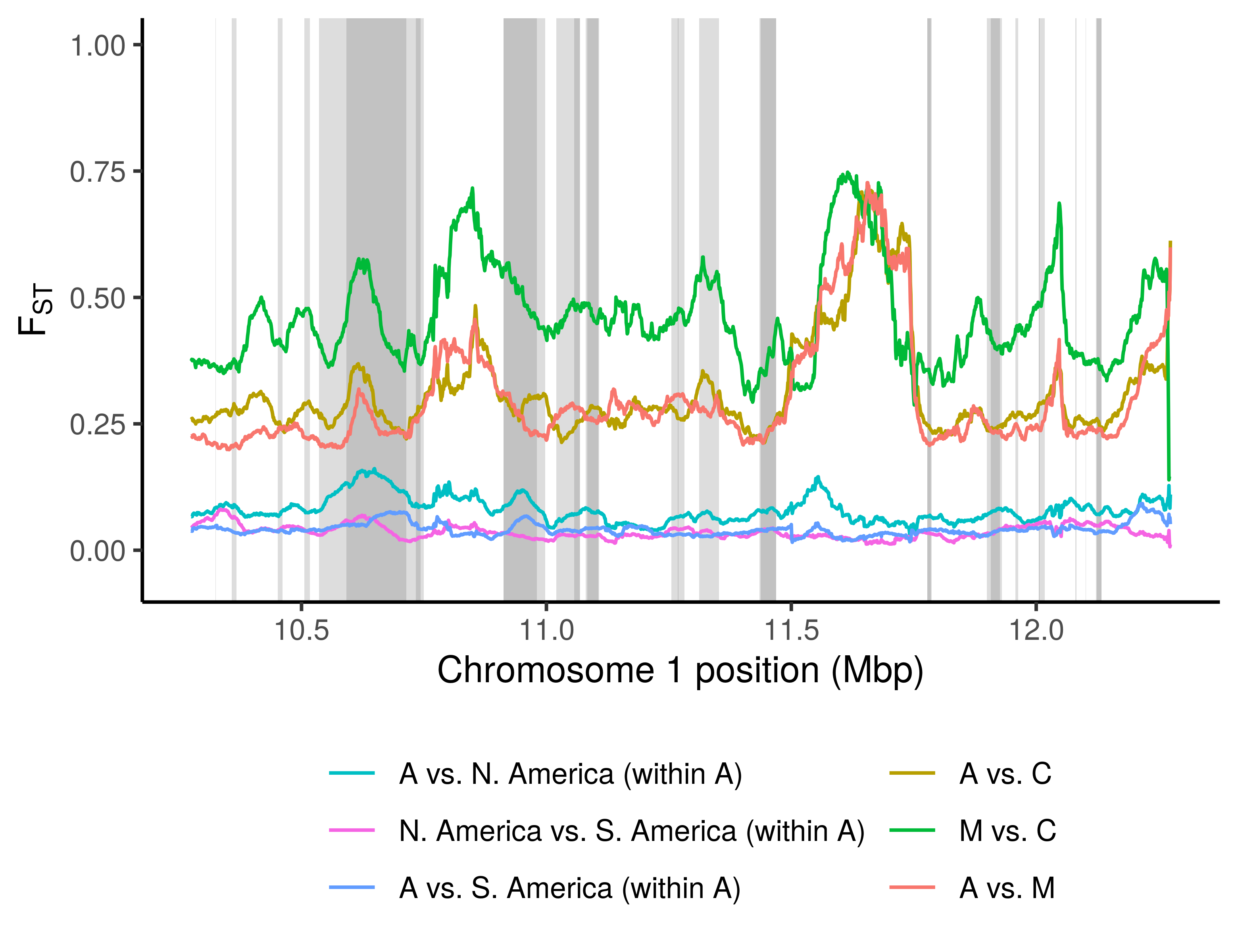

Supplement: S22 Fig — FST across the region on chromosome 1 with shared high A ancestry outliers. Outlier regions meeting a 10% FDR in both hybrid zones are highlighted in darker grey, while those meeting a 10% FDR in only one hybrid zone are highlighted in lighter grey. Per-SNP FST is averaged within sliding 50kb windows. In addition to the three ancestry reference panels (A, C, & M), we include contrasts for the subset of individuals in each hybrid zone with high-confidence homozygous A ancestry. (TIF) [file pgen.1009038.s028.tif]

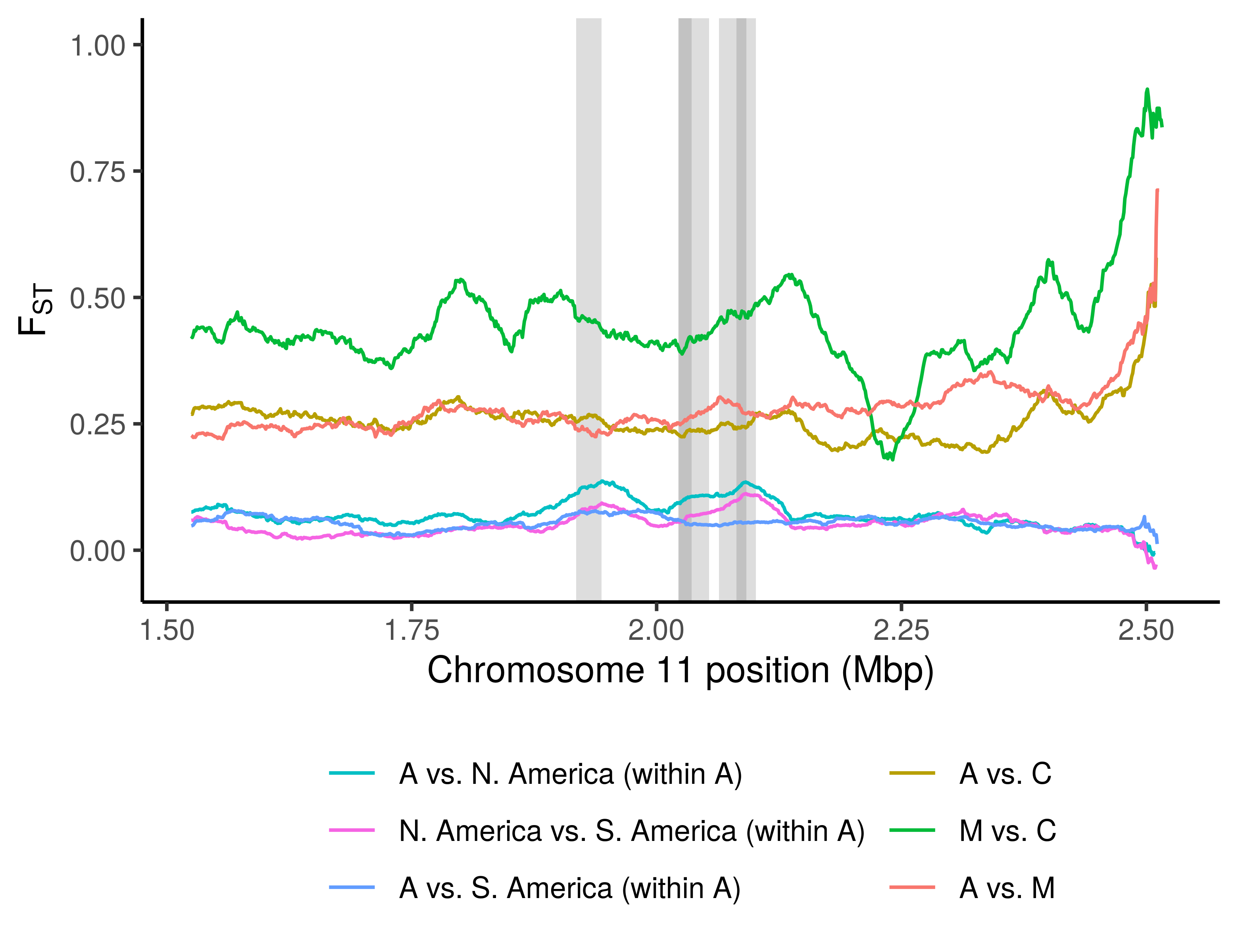

Supplement: S23 Fig — FST across the region on chromosome 11 with shared high A ancestry outliers. Outlier regions meeting a 10% FDR in both hybrid zones are highlighted in darker grey, while those meeting a 10% FDR in only one hybrid zone are highlighted in lighter grey. Per-SNP FST is averaged within sliding 50kb windows. In addition to the three ancestry reference panels (A, C, & M), we include contrasts for the subset of individuals in each hybrid zone with high-confidence homozygous A ancestry. (TIF) [file pgen.1009038.s029.tif]

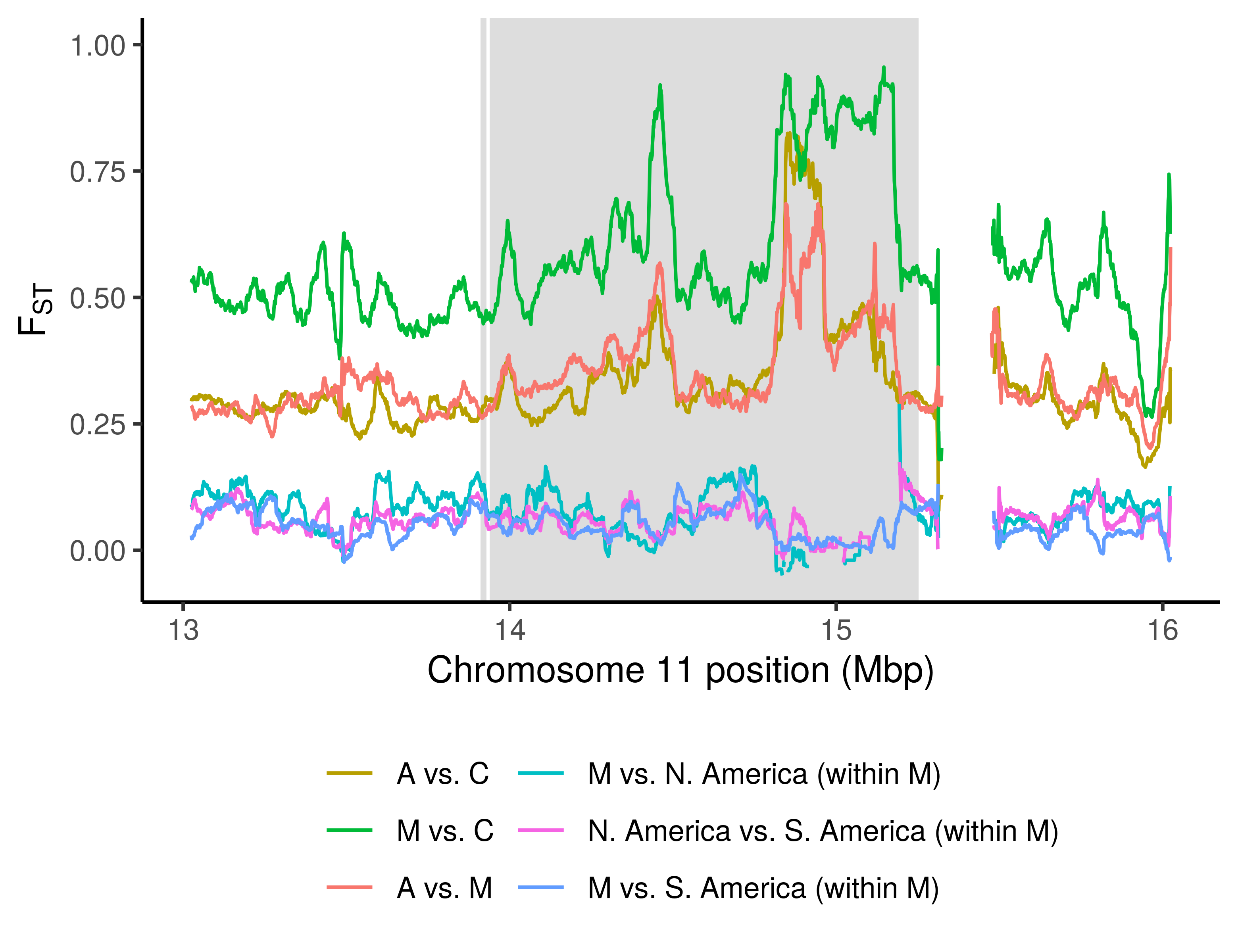

Supplement: S24 Fig — FST across the 1.4Mb region on chromosome 11 with high western European ancestry (M) in South America but not in North America (left), with the outlier region meeting 10% FDR highlighted in grey. Per-SNP FST is averaged within sliding 50kb windows. In addition to the three ancestry reference panels (A, C, & M), we include contrasts for the subset of individuals in each hybrid zone with high-confidence homozygous M ancestry. Windows are dropped if fewer than 10 SNPs have 2 individuals with data, which produces gaps in the contrasts with N. American M ancestry because this hybrid zone does not have elevated M ancestry in this region and at many SNPs very few individuals have high-confidence homozygous M ancestry. Top peaks in M vs. C, M vs. A, and C vs. A contrasts seen within this region reach the 99.5, 98.0, and 99.8 percentiles (respectively) for 50kb windows genome-wide. (TIF) [file pgen.1009038.s030.tif]

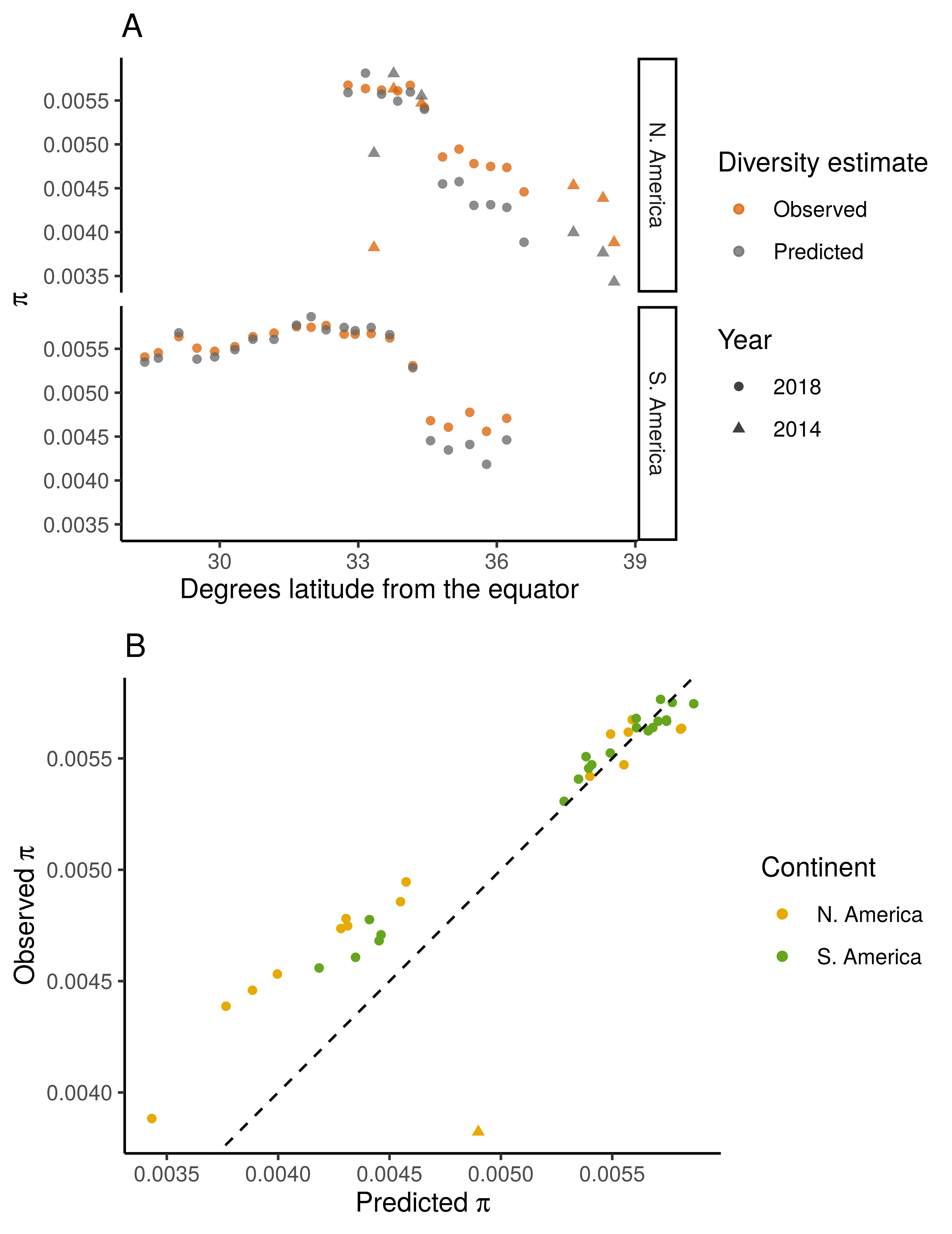

Supplement: S25 Fig — (A) Observed and predicted allelic diversity (π) for each population across latitude. To predict π for a specific population, we calculated the expected allele frequency based on a mixture of A, C, and M reference population allele frequencies, weighted by the population’s estimated admixture fractions of these three ancestries. (B) Plot of predicted vs. observed π for each population for direct comparison to the 1-to-1 line (dashed). Avalon (California 2014, marked as a triangle) is a clear outlier, with low diversity for its admixture fraction. (TIF) [file pgen.1009038.s031.tif]

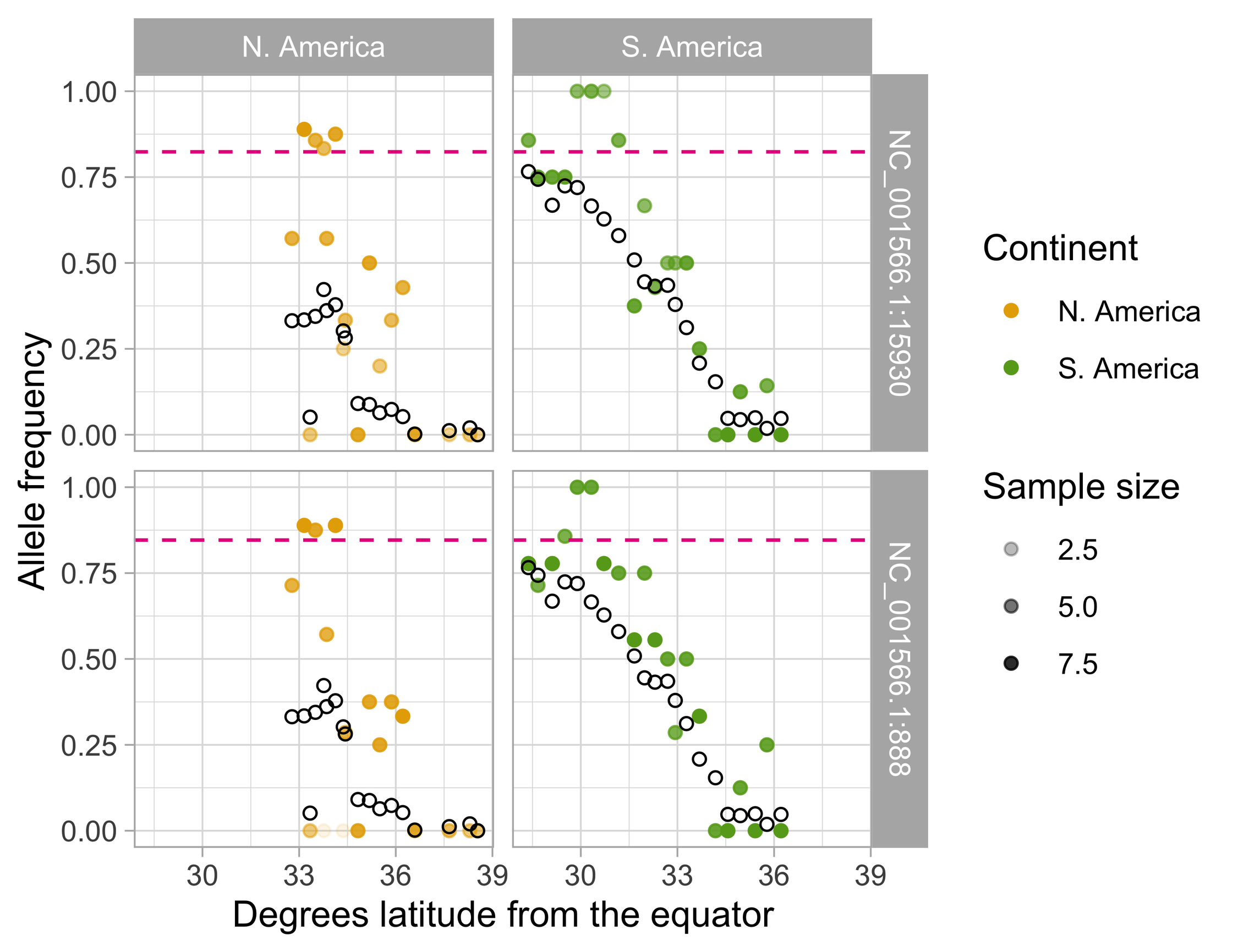

Supplement: S26 Fig — Out of 82 SNPs on the mitochondria, we identified two with more than 80% estimated frequency difference between scutellata (A) and European (C & M) reference panels. Estimated allele frequencies at these SNPs for each population in North America (left) and South America (right) are plotted in color. For comparison, population mean genomewide A ancestry proportions (NGSAdmix) are plotted as open black circles. At both SNPs, estimated M and C allele frequencies are zero (not shown) and estimated A allele frequencies are high but not at fixation (plotted as pink dashed lines). Bees sequenced in this study have low coverage across most of the mtDNA sequence, which prevented us from constructing a phylogenetic tree for full mitochondrial haplotypes and creates uncertainty in our allele frequency estimates here. To reflect this uncertainty, points are shaded by the sample size (i.e. the number of mtDNA haplotypes in a population for which we were able to call a consensus base). (TIF) [file pgen.1009038.s032.tif]
