# Supplementary material for: Selection and hybridization shaped the rapid spread of African honey bee ancestry in the Americas
Source: PLoS Genet. 2020 Oct 19;16(10):e1009038. doi: 10.1371/journal.pgen.1009038 (PMC7595643; doi:10.1371/journal.pgen.1009038)
Supplement: Attachment — Submitted filename: response_to_reviewers_7.31.2020.pdf [file pgen.1009038.s033.pdf]

Dear PLOS Genetics,
Thank you for the thoughtful reviews. Our responses to individual reviewer comments are in-line below. We have changed the terminology in our paper from 'Africanized honey bees' to the more scientifically accurate '*scutellata*-European hybrid honey bees' and describe these changes below the response to reviews. We also include below our response to a question from the PLOS office as well as changes in response to other feedback. We have attached both a marked and clean copy of the revised paper for your review. We hope you agree that these changes have improved the manuscript and we are looking forward to hearing back from you.
Sincerely,
Erin Calfee

Reviewer Comments and Proposed Changes:
Reviewer #1: In this MS, Calfee and colleagues present a well-crafted and careful study of the genomics of Africanized honey bee invasion in the Americas. The authors demonstrate quite convincingly that the invasion has likely reached some climactic limit, and that adaptive traits of the invasive bees are broadly polygenic. The authors find several candidate regions in which ancestry proportions and clines deviate significantly from the remainder of the genome, and give plausible selection scenarios to explain these regions. All in all I found the paper anticipated potential issues for most results very well, and does a great job of conservatively interpreting the results of its many analyses.

Thank you for your review!

Major Comments:

I think the only place where I desired a bit more of an in-depth look was in looking at ancestry correlations at the extreme ends of the clines (Figures 3, S14,S15). The authors present one potential explanation in the form of convergent selection, and suggest the pattern is not driven by any particular region, but Figure S14 seems to show a distribution of Cold NA vs Cold SA driven largely by chromosome 1.

Chromosome 1 does give the strongest signal because of the large cluster of high A ancestry outlier loci shared between continents. We repeated the analysis from Fig S14 dropping both chromosomes with large outlier regions (chromosome 1 and chromosome 11) and the same conclusions hold (see Fig S14).

We have also added a new supplemental figure showing the ancestry covariance matrix directly, instead of the ancestry correlation matrix (see new supplemental Fig S16). Because populations near the cline center have higher ancestry variance (expected due to ancestry proportions closer to 50%), this gives a slightly different picture. However, using covariances we still see excess affinity between the tails of these two hybrid zones, which is unexpected under simple spread. Under a neutral model, both 'low A' North American and 'low A' South American populations should be more similar to geographically closer 'high A' South American populations than they are to each other.

Note: Based on a minor suggestion from reviewer #1, we have changed our population groupings here from 'cold'/'warm' to 'low A'/'high A' (see below).

Additionally, it's not clear to me how convergent selection could drive ancestry correlations to be higher between NA and SA samples than they are within the cold SA samples.
This may simply be a result of heterogeneity within the ‘low A’ South American samples, which vary in their climatic similarity and ancestry correlations to each other and NA. Our division of populations into broad geographic regions is based on a somewhat arbitrary cutoff at the cline center. We have added a legend for ‘Low A’ and ‘High A’ to Fig 3 to highlight the heterogeneity within these groupings.

One potential driver I could think of would be recent shared ancestry between honey bees from both NA and SA, perhaps due to human trading. I would love to a see a genome-wide gene tree/network for all of the samples to see if there is some evidence for some clustering there as well. In general, I think a gene tree of your samples, while not essential, would be helpful in aiding readers interpret some of the results.

Thank you for suggesting additional analyses to strengthen these results. We have done more work to assess the possibility of (human mediated) long distance migration between the two ends of the hybrid zone. Because populations at the two high-latitude ends for the clines have similar ancestry compositions, we would expect them to cluster in any genetic relatedness analysis that ignores ancestry (e.g. PCA or phylogenetic trees) even without gene flow. To get around this limitation, we identify high-confidence homozygous ancestry tracts and use within-ancestry covariances and PCAs to assess evidence of recent kinship for each ancestry separately. Recent gene flow between the two tails of the hybrid zones should lead to excess allelic covariances within each ancestry type, which we do not see (see new Figs S17-19). We have added these analyses to the methods in a new subsection (“Ancestry correlations between the high-latitude cline endpoints”) and reference them in the results: “Another possible explanation is recent long-distance migration (e.g. international bee exports); however, we investigated genetic covariance patterns within A, C, and M ancestries and found no clear evidence of gene flow between the high-latitude cline endpoints (see methods).”

Minor Comments:

"Cold vs Warm" terminology: I found this terminology both confusing and not very useful as the two parts of the cline are determined by ancestry and not by climate. I understand that there *are* broad climactic differences as well, but perhaps "High A" vs "Low A" or similar could be used, as I found myself thinking purely in terms of climate and not ancestry.

We agree and have changed our labels to "High A" and "Low A" in the text and supplementary figures (Figs S14 and S15). We have also added these labels in a legend to Fig 3. We now define these groups directly in terms of the geography of the cline, which we hope is more intuitive (see methods “Ancestry correlations between the cline endpoints”). We also added this explanatory text to the Fig 3 caption: “We defined low and high A ancestry regions for each hybrid zone based on the estimated latitude for the genome-wide cline center. Roughly half of the sampled South American populations, and all of the North American populations, fall in the 'low A' half of their respective hybrid zones.”

Line 140: you should define b, not w, here, especially since you don't seem to use w in the rest of the main text.

We would like to keep the definition of w because it makes it easier to compare our work to the historical literature on clines and because cline width (unlike b) has a physical interpretation. Thank you for pointing out that the relevance of cline width was unclear. We have added a parenthetical explanation of cline width after it's definition in relation to b: "(i.e. the inverse of the steepest gradient at the center of the cline)".

Figure 7: Add label for the genome wide patterns similar to the two outlier SNP categories.
We have added a label for the genomewide mean to the Fig 7 legend as suggested.

Line 428: Purely stylistic - I thought I had missed some big results about wing length with that introduction. I completely agree that admixture mapping is potentially extremely powerful in this system, but perhaps just say that as you have not found any significant results to serve as an example.
We agree that this emphasis on admixture mapping was misleading given our null result and we have removed this paragraph from the discussion.

Reviewer #2: Review of "Selection and hybridization shaped the Africanized honey bee invasion of the Americas"

In this paper, the authors explore the spread of African honey bee ancestry in North and South America. They sampled and sequenced populations along transects spanning dual hybrid zones at either end of Africanized bee ancestry. The authors find that ancestry and wing size clines are surprisingly consistent in both continents, although they also highlight several ancestry outlier regions, suggesting the action of selection.

This is a very, very good paper. I've never actually done this before, but I don't have any suggestions or critiques. This paper succeeds at two levels. On the topic of africanized honey bees, the finding that the hybrid zone is consistent between continents is really important for understanding what limits the spread of africanized ancestry. The authors also find several regions that look like they could be under selection for different ancestry groups, which are intriguing now and could turn up important features of bee biology if probed further. At an evolutionary biology level, this paper is a great example of how selection can create the bounds of a hybrid zone. The authors also do an amazing job of just being very careful and thorough. I really appreciate the effort to simulate realistic null distributions. Altogether, this paper is a great example of how to do a population genomics analysis. I commend the authors.

Thank you for your review!

Minor note:
Line 545: "…bees sequenced in y…" is that correct?
This was a typo that should read "bees sequenced in this study". We have corrected the text.

PLOS CC-BY Maps
In response to the concern from PLOS that all maps must follow CC-BY, we added text (and citations) to the Fig 1 caption to clarify that the maps were created by the authors using public data and open-source software: "The underlying maps were created by plotting geographic data from the CIA World DataBank II in R using ggplot."

Change of Scientific Terms

In the original submission we used the historical term ‘Africanized honey bees’ but have reconsidered this choice because it may mislead readers about the percent of A ancestry in hybrid bee populations (which spans from > 80% to < 5%) and risks misrepresenting what we know about other African honey bees subspecies (that do not all share *scutellata*-like traits). We have therefore switched to more precise scientific language. When talking about the invasion and hybrid populations we have replaced ‘Africanized honey bees’ with ‘*scutellata*-European hybrid honey bees.’ When describing ancestry patterns, we now use ‘*scutellata*’ or simply ‘A’ ancestry in place of ‘African’ ancestry throughout the text and figures. We also think that using these terms can help avoid sensationalization of honey bees, particularly in the popular media, where terms like ‘Africanized bees’ and ‘killer bees’ are often used interchangeably and assumed to be discrete categories instead of exhibiting continuous variation. This change of terms also necessitated a change of title and, in citing previous studies of *scutellata*-European hybrid honey bees, more precise language about the ancestry composition (or by proxy geographic origin) of the bees studied. When introducing the invasion, we now also describe more of the geographic and historical context for both *scutellata* and European honey bee introductions to the Americas. We explain what is known about the ancestry composition of *scutellata*-European hybrid honey bees, and our reason for using new terms, in a new paragraph of the introduction:

“Previous genomic work on the invasion has shown that *scutellata*-European hybrid honey bees are a genetic mixture of three major genetic groups: A from Africa, C from eastern Europe and M from western Europe. Historical sources indicate that the A ancestry is from *A. m. scutellata*, while both M and C ancestries are mixtures of multiple subspecies imported from Europe, e.g. *A. m. ligustica* (C), *A. m. carnica* (C), *A. m. mellifera* (M), and *A. m. iberiensis* (M). Many names have been used previously to refer to *scutellata*-European hybrids in the literature, including ‘African honey bees’, ‘African hybrid honey bees’, or ‘Africanized honey bees’, and the ambiguous acronym ‘AHB’, with these names being used to describe bees identified as having *scutellata* ancestry on the basis of behavior, morphology, mtDNA, or a range of *scutellata* autosomal ancestry. Given the wide range of *scutellata*-European ancestry that we find in this study, and that *A. m. scutellata* is only one of at least 10 ecologically diverse *Apis mellifera* subspecies native to Africa, we will simply use the label *scutellata*-European hybrids for individuals whose autosomal genome is comprised of a mixture of these ancestries.”

Other Changes
In response to feedback from other readers, we made the additional changes listed below by section.

Abstract:
We removed ‘remarkable’ from the first sentence.

Changed ‘further’ to ‘farther’.

We separated our two main results on individual ancestry clines into two sentences: “Ancestry clines at individual SNPs are consistent with a broad genetic basis for climate-associated fitness trade-offs, with a build-up of somewhat steeper clines in regions of low recombination and no large effect loci maintaining exceptionally steep ancestry transitions.” -> “We find no large effect loci maintaining exceptionally steep ancestry transitions. Instead, we find most individual loci have concordant ancestry clines across

South America, with a build-up of somewhat steeper clines in regions of the genome with low recombination rates, consistent with many loci of small effect contributing to climate-associated fitness trade-offs."

We added 'on either continent' to distinguish diversity results that apply to both hybrid zones from the individual locus cline results that only apply to South America (where we observed the full cline).

Changed 'broad genetic' to 'polygenic'.

Author summary:
Changed 'widely successful' to 'ecologically successful'.

Introduction:
We added citations for two relevant recent papers, on hybrid incompatibilities (Powell et al. 2020) and adaptive introgression (Bay et al. 2019), to the introduction.

We added a small paragraph describing the defensive behavior of *scutellata*-European hybrid honey bees and acknowledge that this behavior has been sensationalized by terms such as 'killer bees', with new citations.

We moved the sentence about the recent genomic study in Brazil up to the paragraph giving background on the genetic basis of high fitness in *scutellata*-European hybrid honey bees: "European ancestry may have also contributed to the success of the invasion; a recent study of *scutellata*-European hybrid bees in Brazil revealed some European alleles at exceptionally high frequency, but this work was under-powered to detect high-fitness *scutellata* alleles due to elevated genome-wide *scutellata* ancestry (84%) in the Brazilian population."

We removed colony defense from the list of traits that may have given *scutellata*-European honey bees a selective advantage and have moved this trait to a new paragraph on traits with less predictable relationships to fitness.

Results:

Fig 1 we quantified transect distance in the caption: "Sampling spanned 646km in California and 878km in Argentina in the north-south direction." Also added text to contextualize outliers: "These earlier California samples include one island population, Avalon (Catalina Island), indicated by a yellow triangle. Bees from Avalon have majority M ancestry, in contrast to all mainland California bees which have predominantly A and C ancestry."

Added a sentence to clarify that timing of admixture was jointly estimated with local ancestry tracts using ancestry_hmm: "For each population, we applied a hidden Markov model that jointly infers the maximum likelihood single-pulse approximation for the generations since mixture and posterior probabilities for local ancestry state, based on read counts from low-coverage sequence data."

Deleted extra word ('and') from original line 123.

Fig 2 we changed the shape of outlier points from Avalon and added the text "Samples from Avalon are displayed as orange triangles; Catalina Island has a distinct ancestry

composition from mainland California populations and low A ancestry for its latitude." We also expanded the range of the y-axis for the lower wing length plot because some points were falling outside of the plot and not being properly displayed.

We have reworded the results section for wing lengths to be more cautious about interpreting this cline as ancestry-based and we have added a relevant historical citation: “Genetic crosses have shown that wing length differences between ancestries have a genetic basis and the wing length patterns we observe here are consistent with expectations of an additive polygenic cline based on genome-wide ancestry alone (Fig 2). However, the phenotypic clines could alternatively be caused purely by developmental plasticity or sorting of within-ancestry genetic variation along a latitudinal gradient. Preliminary evidence that other factors may contribute to the wing length clines observed here comes from a 1991 survey showing that wing length was positively correlated with latitude in California’s feral bee populations before the reported arrival of *scutellata*-European hybrid honey bees. From field-based sampling alone, it remains unclear what portion of the observed phenotypic clines are ancestry-driven.”

Changed ‘observed’ ancestry to ‘inferred’ ancestry.

Added parenthetical text to justify the use of non-linear least squares over other estimation methods (e.g. maximum likelihood). For genomewide clines: “(although maximum likelihood or Bayesian estimation are generally preferred when the errors can be fully parameterized, here least squares allows for unknown drift variance in addition to binomial sampling variance).” For individual loci: “Similar cline models have been fit using likelihood methods under some simplifying assumptions about the form of the errors (e.g. citations). We instead use non-linear least squares to fit cline parameters without specifying a full error model and then quantify the effects of more complex unmodeled errors (including ancestry variances and covariances) through simulation.”

Changed multiple instances of ‘SNPs’ to ‘loci’ in the text where the theory we propose is more general to any type of locus. Also defined ‘SNP’ at first use.

Clarified ambiguity in the text describing our simulations. By ‘unlinked loci’ we mean independent loci, i.e. independent draws from the multivariate-normal distribution: “We simulated data for 100,000 independent loci undergoing drift, which we used to estimate the expected distribution of neutral clines and calculate false-discovery rates. For each simulated locus, we independently drew a vector of population ancestry frequencies from a multivariate-normal model of drift, ...”

We added text to clarify why we only include South America in the cline analysis and why we use multiple approaches for detecting outlier loci: “We limit the analysis of clines at individual loci to South America where, unlike North America, we have samples spanning both halves of the hybrid zone to inform parameter estimates. While cline analyses can be used to identify both adaptive introgression and barriers to introgression (by analysing cline center displacement in addition to cline steepness), here we focus on barrier loci and approach identifying positively selected loci using alternative methods that can be applied to both hybrid zones (see “Scan for ancestry-associated selection”).”

Corrected the mean value for individual SNP clines in South America to 960km, or w = 8.65 (was originally reported as 937km wide, w = 8.44 degrees latitude which is the mean across 0.2cM windows, not SNPs).

We revised the text to emphasize the roles of selection and recombination in maintaining steep clines at selected loci because the original phrasing (‘loci maintaining steeper clines will co-localize’) connoted undue agency for the loci: “Under a polygenic climate barrier, we expect locally-adapted loci to be found across the genome but steeper clines to be more commonly maintained in regions with low recombination rates. This is because selected loci create stronger barriers to gene flow when there is tight genetic linkage than when selection acts on each locus independently.”

Corrected typo that posterior threshold is > 0.8 (not greater than or equal to 0.8) for calling high-confidence ancestry tracts; now this is consistent throughout the text.

Some readers found the original Fig 5 hard to interpret. We moved the axes ticks to the left/bottom and added a grid to the plot background so that it is more clear that the marginal histogram and main scatterplot share the same axes/units. In the figure caption, we added this information, re-arranged some of the original text, changed ‘hex’ to ‘hexagon’, ‘circle’ to ‘ellipse’ and added a note about the streaky appearance of the scatterplot: “Note: While SNPs are thinned for LD, large outlier regions span many SNPs, which creates the streak-like patterns in the scatterplot.” We also added additional text to the Fig 5 caption to more quantitatively describe the fit of the data to the null model: “Most of the genome is consistent with neutrality under a MVN normal model of drift (98.6% of SNPs fall within the orange ellipse), but there are also some clear outliers.”

Discussion:

In the discussion, we now additionally cite the Drosophila literature on ‘continuous-environment clines’ when describing other systems with broad adaptive clines: “These proposed dynamics are similar to well-studied cases in other systems where bounded hybrid superiority and/or local adaptation to continuous environments maintain adaptive clines across broad geographic regions.”

Changed ‘broad genetic’ to ‘polygenic’.

We added a new paragraph to the discussion expanding on our conclusion that *scutellata*-European hybrid honey bees cannot be treated as a cohesive group. Given that a broad audience (e.g. beekeepers) is interested in new scientific findings on this topic, we have used this paragraph to discuss the limitations of ‘Africanized’ honey bee identification (e.g. via genetics) and using ancestry as a proxy for trait screening (e.g. for defense).

Methods:
We added a sentence with citations for key software: “Statistical results and figures were created in R (cited) with use of the tidyverse (cited) packages. Other scripts were run using GNU parallel (cited).”

line 340: Added version number to ancestry_hmm reference in methods - v0.94

Citations:

We added citations to existing text for bedtools, samtools, Benjamini-Hochberg corrected FDRs, and R packages nls.multstart (cline fits), geosphere (geographic distances), poibin (Poisson binomial dist.) and MASS (multivariate normal dist.).

Data Availability:
Added GitHub repository information: “Scripts are available at https://github.com/ecalfee/bees.”

Acknowledgements:
We added acknowledgements for additional colleagues who provided feedback (see acknowledgements).

Supplementary Figures:
Rescaled Fig S10 (shorter y axis) for better visualization of the distribution of individual SNP clines.
